# Supplementary material for: Association of late-onset postpartum depression of mothers with expressive language development during infancy and early childhood: the HBC study
Source: PeerJ. 2019 Mar 6;7:e6566. doi: 10.7717/peerj.6566 (PMC6408909; doi:10.7717/peerj.6566)
Supplement: Supplemental Information 1 — Only six non-Japanese children were included in this cohort. aNot all participants attended all follow-up visits planned. b Z-scores for which the mean is 0 and standard deviation (sd) is 1. [file peerj-07-6566-s001.docx]

|  |  | No PPD  (n=823, 84.9%) | Early-onset PPD  (n=103, 10.6%) | Late-onset PPD  (n=43, 4.4%) |
| --- | --- | --- | --- | --- |
|  |  | n (%) or Mean (sd) | n (%) or Mean (sd) | n (%) or Mean (sd) |
| Age in month of the child^a^ | |  |  |  |
|  | At 10 months (n=936) | 10.5 (0.5) | 10.5 (0.5) | 10.6 (0.7) |
|  | At 14 months (n=879) | 14.5 (0.6) | 14.5 (0.6) | 14.7 (0.7) |
|  | At 18 months (n=924) | 18.6 (0.7) | 18.8 (0.9) | 18.6 (0.6) |
|  | At 24 months (n=912) | 24.7 (0.9) | 24.8 (1.1) | 25.0 (1.1) |
|  | At 32 months (n=883) | 33.2 (2.0) | 33.5 (1.7) | 33.1 (1.4) |
|  | At 40 months (n=884) | 39.8 (2.2) | 40.2 (2.3) | 39.6 (1.2) |
| Infant sex | |  |  |  |
|  | male | 420 (51%) | 47 (46%) | 24 (56%) |
| Birth order | |  |  |  |
|  | first-born | 394 (47%) | 69 (67%) | 22 (51%) |
|  | second-born | 318 (39%) | 29 (28%) | 16 (37%) |
|  | third-born or later | 111 (14%) | 5 (5%) | 5 (12%) |
| Multiparity | |  |  |  |
|  | twin birth | 22 (3%) | 2 (2%) | 6 (14%) |
| Birthweight (g) | | 2955 (438) | 2882 (427) | 2880 (413) |
| Gestational age at birth (in weeks) | | 39.0 (1.5) | 39.1 (1.4) | 38.7 (1.4) |
| Duration of breastfeeding (in months) | | 10.0 (6.1) | 8.6 (6.3) | 9.3 (6.0) |
| Maternal history of mood disorders | | 67 (8%) | 25 (24%) | 7 (16%) |
| Maternal history of anxiety disorders | | 27 (3%) | 7 (7%) | 1 (2%) |
| Maternal age at the infant’s birth (in years) | | 31.7 (5.0) | 31.5 (5.0) | 32.7 (5.5) |
| Maternal education level (in years) | | 14.0 (1.9) | 13.8 (2.0) | 14.2 (1.9) |
| Paternal age at the infant’s birth (in years) | | 33.6 (5.7) | 32.8 (5.7) | 34.5 (6.9) |
| Paternal education level (in years) | | 14.3 (2.7) | 13.9 (2.4) | 14.1 (2.5) |
| Annual household income at birth (million yen) | | 6.2 (3.0) | 5.9 (2.3) | 5.5 (1.5) |
| Edinburgh Postnatal Depression Scale | |  |  |  |
|  | Score at 1st measurement [at the 2nd week] | 2.8 (2.2) | 11.7 (3.0) | 5.7 (1.5) |
|  | Score at 2nd measurement [at the 4th week] | 2.2 (2.1) | 8.3 (5.1) | 7.8 (4.3) |
|  | Score at 3rd measurement [between 5th and 12th weeks] | 1.8 (2.0) | 6.1 (5.2) | 8.7 (4.1) |
| Expressive Language score^a,b^ | |  |  |  |
|  | At 10 months (n=936) | -0.20 (0.96) | -0.09 (0.99) | -0.25 (1.04) |
|  | At 14 months (n=879) | 0.00 (1.05) | -0.10 (1.05) | -0.27 (1.05) |
|  | At 18 months (n=924) | -0.13 (0.94) | -0.06 (0.97) | -0.60 (1.19) |
|  | At 24 months (n=912) | -0.03 (0.98) | 0.02 (0.98) | -0.72 (1.04) |
|  | At 32 months (n=883) | -0.05 (1.00) | -0.04 (0.89) | -0.62 (1.07) |
|  | At 40 months (n=884) | 0.05 (1.02) | -0.10 (1.01) | -0.64 (1.10) |
