# Supplementary material for: Association of late-onset postpartum depression of mothers with expressive language development during infancy and early childhood: the HBC study
Source: PeerJ. 2019 Mar 6;7:e6566. doi: 10.7717/peerj.6566 (PMC6408909; doi:10.7717/peerj.6566)
Supplement: Supplemental Information 3 — Explanations of the each variable in the dataset, together with the range, missingness, tabulated data reported. [file peerj-07-6566-s003.pdf]

```
--------------------------------------------------------------------------------------------------------------------------
      name:  <unnamed>
       log:  C:¥Kjt¥__Text¥18097Paper_Peerj_Aoyagi¥181031codebook.log
  log type:  text
 opened on:  31 Oct 2018, 10:45:53

. codebook

--------------------------------------------------------------------------------------------------------------------------
id                                                                                       Child's ID (4digits, numeric)
--------------------------------------------------------------------------------------------------------------------------

                  type:  numeric (float)

                 range:  [1,969]                      units:  1
         unique values:  969                      missing .:  0/969

                  mean:       485
              std. dev:   279.871

           percentiles:        10%       25%       50%       75%       90%
                               97       243       485       727       873

--------------------------------------------------------------------------------------------------------------------------
male                                                                              Gender of the child 0/female 1/male
--------------------------------------------------------------------------------------------------------------------------

                  type:  numeric (byte)
                 label:  labmale

                 range:  [0,1]                        units:  1
         unique values:  2                        missing .:  0/969

            tabulation:  Freq.   Numeric  Label
                           478         0  Female
                           491         1  Male

--------------------------------------------------------------------------------------------------------------------------
bw                                                                                                          Birthweight
--------------------------------------------------------------------------------------------------------------------------

                  type:  numeric (float)

                 range:  [946,4286]                   units:  1
         unique values:  558                      missing .:  0/969
```

```
                 mean:   2943.94
             std. dev:    436.22

          percentiles:        10%       25%       50%       75%       90%
                             2406      2676      2964      3226      3480

------------------------------------------------------------------------------------------------------------------------
epdsw2                                                                          Elapse weeks of 1.EPDS at 2W
------------------------------------------------------------------------------------------------------------------------

                 type:  numeric (float)

                range:  [.71,4.71]                   units:  .01
        unique values:  24                       missing .:  2/969

                 mean:   2.27746
             std. dev:     .3731

          percentiles:        10%       25%       50%       75%       90%
                             1.99      2.14      2.14      2.28      2.71

------------------------------------------------------------------------------------------------------------------------
epdsw4                                                                          Elapse weeks of 2.EPDS at 4W
------------------------------------------------------------------------------------------------------------------------

                 type:  numeric (float)

                range:  [2.71,11.57]                 units:  .01
        unique values:  41                       missing .:  0/969

                 mean:   4.42364
             std. dev:    .80823

          percentiles:        10%       25%       50%       75%       90%
                             3.99      4.14      4.14      4.42      4.99

------------------------------------------------------------------------------------------------------------------------
epdsw10                                                                        Elapse weeks of 3.EPDS at 10W
------------------------------------------------------------------------------------------------------------------------

                 type:  numeric (float)

                range:  [4.28,19.14]                 units:  .01
        unique values:  48                       missing .:  167/969

                 mean:   8.59077
```

```
          std. dev:   1.06133

       percentiles:        10%       25%       50%       75%       90%
                          7.99      8.14      8.28      8.71      9.42

--------------------------------------------------------------------------------------------------------------------------
faag01                                                                          Father's age at birth (yrs)
--------------------------------------------------------------------------------------------------------------------------

              type:  numeric (float)

             range:  [18.850103,53.431896]        units:  1.000e-06
     unique values:  896                      missing .:  0/969

              mean:   33.5507
          std. dev:   5.76292

       percentiles:        10%       25%       50%       75%       90%
                       26.5681   29.6783    33.295   37.0431   41.1444

--------------------------------------------------------------------------------------------------------------------------
faed01                                                                             Father's education (yrs)
--------------------------------------------------------------------------------------------------------------------------

              type:  numeric (byte)

             range:  [9,26]                       units:  1
     unique values:  16                       missing .:  0/969

              mean:   14.2508
          std. dev:   2.63677

       percentiles:        10%       25%       50%       75%       90%
                            12        12        14        16        18

--------------------------------------------------------------------------------------------------------------------------
ga                                                                                Gestational age at birth
--------------------------------------------------------------------------------------------------------------------------

              type:  numeric (float)

             range:  [30.139999,42.139999]        units:  1.000e-06
     unique values:  74                       missing .:  0/969

              mean:   38.9673
          std. dev:   1.49545
```

```
         percentiles:        10%       25%       50%       75%       90%
                           37.28     38.14     39.14        40     40.71

------------------------------------------------------------------------------------------------------------------------
hbcm10                                                                                Time elapsed 10M in Months
------------------------------------------------------------------------------------------------------------------------

                type:  numeric (float)

               range:  [9.2,13.41]                  units:  .01
       unique values:  86                       missing .:  33/969

                mean:   10.4529
            std. dev:     .4855

         percentiles:        10%       25%       50%       75%       90%
                           10.02     10.15     10.32     10.65     11.04

------------------------------------------------------------------------------------------------------------------------
hbcm14                                                                                Time elapsed 14M in Months
------------------------------------------------------------------------------------------------------------------------

                type:  numeric (float)

               range:  [12.13,20.64]                units:  .01
       unique values:  96                       missing .:  90/969

                mean:   14.5114
            std. dev:   .640546

         percentiles:        10%       25%       50%       75%       90%
                           14.03     14.13     14.36     14.69     15.18

------------------------------------------------------------------------------------------------------------------------
hbcm18                                                                                 Time elapsed 18M in Month
------------------------------------------------------------------------------------------------------------------------

                type:  numeric (float)

               range:  [16.6,23.2]                  units:  .01
       unique values:  111                      missing .:  45/969

                mean:   18.6149
            std. dev:   .724414
```

```
        percentiles:        10%       25%       50%       75%       90%
                          18.01     18.17     18.44     18.86     19.46

--------------------------------------------------------------------------------------------------------------------------------------
hbcm24                                                                                             Time elapsed 24M in Month
--------------------------------------------------------------------------------------------------------------------------------------

                type:  numeric (float)

               range:  [21.03,33.82]                units:  .01
       unique values:  126                      missing .:  57/969

                mean:   24.7265
            std. dev:   .943821

        percentiles:        10%       25%       50%       75%       90%
                          24.03    24.205     24.49     24.95      25.7

--------------------------------------------------------------------------------------------------------------------------------------
hbcm32                                                                                             Time elapsed 32M in Month
--------------------------------------------------------------------------------------------------------------------------------------

                type:  numeric (float)

               range:  [28.79,59.63]                units:  .01
       unique values:  170                      missing .:  86/969

                mean:   33.2039
            std. dev:   1.96777

        percentiles:        10%       25%       50%       75%       90%
                          31.95     32.28     32.84     33.72     34.81

--------------------------------------------------------------------------------------------------------------------------------------
hbcm40                                                                                             Time elapsed 40M in Month
--------------------------------------------------------------------------------------------------------------------------------------

                type:  numeric (float)

               range:  [32.77,59.63]                units:  .01
       unique values:  200                      missing .:  85/969

                mean:   39.8236
            std. dev:   2.17676

        percentiles:        10%       25%       50%       75%       90%
```

```
                     38.13    38.52    39.41    40.46    41.91

------------------------------------------------------------------------------------------------------
mlelt10                                                             T-score for 10 months [T.Nishimura]
------------------------------------------------------------------------------------------------------

             type:  numeric (float)

            range:  [24.112175,78.673439]       units:  1.000e-06
    unique values:  175                     missing .:  55/969

             mean:   48.1126
         std. dev:    9.6874

      percentiles:        10%       25%       50%       75%       90%
                     36.0021   39.9577   49.4515   55.3192   60.2571

------------------------------------------------------------------------------------------------------
mlelt14                                                             T-score for 14 months [T.Nishimura]
------------------------------------------------------------------------------------------------------

             type:  numeric (float)

            range:  [20,70.431534]            units:  1.000e-06
    unique values:  203                     missing .:  120/969

             mean:    49.147
         std. dev:   10.4845

      percentiles:        10%       25%       50%       75%       90%
                     29.1931   43.6865   51.0702   57.2925   60.0009

------------------------------------------------------------------------------------------------------
mlelt18                                                             T-score for 18 months [T.Nishimura]
------------------------------------------------------------------------------------------------------

             type:  numeric (float)

            range:  [20,76.601952]            units:  1.000e-06
    unique values:  230                     missing .:  71/969

             mean:   48.5835
         std. dev:   9.54355

      percentiles:        10%       25%       50%       75%       90%
                     37.7175   43.1946   49.1099   54.6094   60.5194
```

```
--------------------------------------------------------------------------------------------------------------
mlelt24                                                              T-score for 24 months [T.Nishimura]
--------------------------------------------------------------------------------------------------------------

              type:  numeric (float)

             range:  [20,80]                   units:  1.000e-06
       unique values:  294                   missing .:  95/969

              mean:   49.4513
          std. dev:   9.93504

         percentiles:       10%       25%       50%       75%       90%
                         36.4195   44.6124   50.2865   56.2504   60.6349

--------------------------------------------------------------------------------------------------------------
mlelt32                                                              T-score for 32 months [T.Nishimura]
--------------------------------------------------------------------------------------------------------------

              type:  numeric (float)

             range:  [20,80]                   units:  1.000e-06
       unique values:  410                   missing .:  120/969

              mean:   49.2762
          std. dev:   9.94061

         percentiles:       10%       25%       50%       75%       90%
                          37.692   42.8155   49.1079   55.7562   61.5146

--------------------------------------------------------------------------------------------------------------
mlelt40                                                              T-score for 40 months [T.Nishimura]
--------------------------------------------------------------------------------------------------------------

              type:  numeric (float)

             range:  [20,78.538284]            units:  1.000e-06
       unique values:  492                   missing .:  110/969

              mean:   50.0184
          std. dev:   10.2811

         percentiles:       10%       25%       50%       75%       90%
                         36.9803   43.8094   49.8049   56.0404   63.3295
```

```
------------------------------------------------------------------------------------------------------------------------
moag01                                                                           Mother's age at child's birth
------------------------------------------------------------------------------------------------------------------------

               type:  numeric (float)

              range:  [17.735798,44.555782]       units:  1.000e-06
       unique values:  901                  missing .:  0/969

               mean:   31.7459
           std. dev:   5.02419

        percentiles:        10%       25%       50%       75%       90%
                       25.1417   28.2081   31.8686   35.3895   38.2122

------------------------------------------------------------------------------------------------------------------------
moed01                                                                    Mother's educational history (yrs)
------------------------------------------------------------------------------------------------------------------------

               type:  numeric (byte)

              range:  [9,23]                    units:  1
       unique values:  14                   missing .:  0/969

               mean:   13.9494
           std. dev:   1.91517

        percentiles:        10%       25%       50%       75%       90%
                            12        12        14        16        16

------------------------------------------------------------------------------------------------------------------------
mopd01                                                                                  Mother's EPDS at 2w
------------------------------------------------------------------------------------------------------------------------

               type:  numeric (float)

              range:  [0,22]                    units:  1
       unique values:  21                   missing .:  2/969

               mean:    3.8635
           std. dev:   3.56455

        percentiles:        10%       25%       50%       75%       90%
                             0         1         3         5         9

------------------------------------------------------------------------------------------------------------------------
```

```
mopd02                                                                               Mother's EPDS at 4w
--------------------------------------------------------------------------------------------------------------------------

                type:  numeric (float)

               range:  [0,21]                      units:  1
       unique values:  21                      missing .:  0/969

                mean:   3.05986
            std. dev:   3.44264

         percentiles:        10%       25%       50%       75%       90%
                              0         1         2         4         7

--------------------------------------------------------------------------------------------------------------------------
mopd3                                                    PPD Early/Late 0/No PPD 1/Early PPD(2-4w) 2/Late PPD(5-12w)
--------------------------------------------------------------------------------------------------------------------------

                type:  numeric (float)
               label:  labppd3

               range:  [0,2]                       units:  1
       unique values:  3                       missing .:  0/969

          tabulation:  Freq.   Numeric  Label
                         823         0  PPD-
                         103         1  PPD_Early
                          43         2  PPD_Late

--------------------------------------------------------------------------------------------------------------------------
mopd03                                                                               Mother's EPDS at 8w
--------------------------------------------------------------------------------------------------------------------------

                type:  numeric (float)

               range:  [0,28]                      units:  1
       unique values:  21                      missing .:  167/969

                mean:   2.59476
            std. dev:   3.23937

         percentiles:        10%       25%       50%       75%       90%
                              0         0         1         4         7

--------------------------------------------------------------------------------------------------------------------------
mopsyafdfor0                                             Maternal LIFETIME Affect Disord BY the index preg (29600-99)
```

```
------------------------------------------------------------------------------------------------------------------------

              type:  numeric (float)
             label:  labny

             range:  [0,1]                    units:  1
     unique values:  2                    missing .:  0/969

        tabulation:  Freq.  Numeric  Label
                       870        0  No
                        99        1  Yes

------------------------------------------------------------------------------------------------------------------------
mopsyafdfor40                                          Maternal LIFETIME Affect Disord during 0-40M (29600-99)
------------------------------------------------------------------------------------------------------------------------

              type:  numeric (float)
             label:  labny

             range:  [0,1]                    units:  1
     unique values:  2                    missing .:  0/969

        tabulation:  Freq.  Numeric  Label
                       819        0  No
                       150        1  Yes

------------------------------------------------------------------------------------------------------------------------
mopsyanxfor0                                Maternal LIFETIME Anxiety Disord BY the index preg (30000/30041:30928)
------------------------------------------------------------------------------------------------------------------------

              type:  numeric (float)
             label:  labny

             range:  [0,1]                    units:  1
     unique values:  2                    missing .:  0/969

        tabulation:  Freq.  Numeric  Label
                       934        0  No
                        35        1  Yes

------------------------------------------------------------------------------------------------------------------------
mopsyanxfor40                                    Maternal LIFETIME Anxiety Disord during 0-40M (30000/30041:30928)
------------------------------------------------------------------------------------------------------------------------

              type:  numeric (float)
             label:  labny
```

```
             range:  [0,1]                    units:  1
     unique values:  2                    missing .:  0/969

        tabulation:  Freq.   Numeric  Label
                       916         0  No
                        53         1  Yes

------------------------------------------------------------------------------------------------------------------------
parit3                                                                Number of children born before 0/0 1/1 2/2+
------------------------------------------------------------------------------------------------------------------------

              type:  numeric (float)
             label:  labparit3, but 2 nonmissing values are not labeled

             range:  [0,2]                    units:  1
     unique values:  3                    missing .:  0/969

        tabulation:  Freq.   Numeric  Label
                       485         0
                       363         1
                       121         2  2+

------------------------------------------------------------------------------------------------------------------------
twin                                                                                  Twins/multiple births
------------------------------------------------------------------------------------------------------------------------

              type:  numeric (float)

             range:  [0,1]                    units:  1
     unique values:  2                    missing .:  0/969

        tabulation:  Freq.  Value
                       939  0
                        30  1

------------------------------------------------------------------------------------------------------------------------
bf                                                                          Duration of breastfeeding in month
------------------------------------------------------------------------------------------------------------------------

              type:  numeric (float)

             range:  [0,23.07]                 units:  1.000e-08
     unique values:  272                  missing .:  0/969

              mean:   9.80104
```

```
           std. dev:   6.16137

         percentiles:        10%       25%       50%       75%       90%
                           1.05      4.37     10.22     14.43     18.37

------------------------------------------------------------------------------------------------------------------------
bf3                                     Duration of breastfeeding in month categorized 0: bf=0, 1: 0<bf<6, 2: bf>=6
------------------------------------------------------------------------------------------------------------------------

                type:  numeric (float)

               range:  [0,2]                     units:  1
       unique values:  3                     missing .:  0/969

          tabulation:  Freq.  Value
                          55  0
                         223  1
                         691  2

. log c
      name:  <unnamed>
       log:  C:¥Kjt¥__Text¥18097Paper_Peerj_Aoyagi¥181031codebook.log
  log type:  text
 closed on:  31 Oct 2018, 10:46:03
------------------------------------------------------------------------------------------------------------------------
```
