# Supplementary material for: Association of late-onset postpartum depression of mothers with expressive language development during infancy and early childhood: the HBC study
Source: PeerJ. 2019 Mar 6;7:e6566. doi: 10.7717/peerj.6566 (PMC6408909; doi:10.7717/peerj.6566)
Supplement: Supplemental Information 5 — Explanations of the each variable in the dataset, together with the range, missingness, tabulated data reported. [file peerj-07-6566-s005.pdf]

```
------------------------------------------------------------------------------------------------------------------------
     name:  <unnamed>
      log:  C:¥Kjt¥__Text¥18097Paper_Peerj_Aoyagi¥181031codebook_long.log
 log type:  text
opened on:  31 Oct 2018, 10:47:10

. codebook

------------------------------------------------------------------------------------------------------------------------
id                                                                                    Child's ID (4digits, numeric)
------------------------------------------------------------------------------------------------------------------------

               type:  numeric (float)

              range:  [1,969]                   units:  1
      unique values:  969                   missing .:  0/5,814

               mean:       485
           std. dev:    279.75

        percentiles:       10%       25%       50%       75%       90%
                            97       243       485       727       873

------------------------------------------------------------------------------------------------------------------------
t                                                                             Elapsed time since childbirth in month
------------------------------------------------------------------------------------------------------------------------

               type:  numeric (byte)

              range:  [10,40]                   units:  1
      unique values:  6                     missing .:  0/5,814

         tabulation:  Freq.  Value
                        969  10
                        969  14
                        969  18
                        969  24
                        969  32
                        969  40

------------------------------------------------------------------------------------------------------------------------
male                                                                             Gender of the child 0/female 1/male
------------------------------------------------------------------------------------------------------------------------

               type:  numeric (byte)
              label:  labmale
```

```
                  range:  [0,1]                    units:  1
         unique values:  2                     missing .:  0/5,814

            tabulation:  Freq.   Numeric  Label
                         2,868         0  Female
                         2,946         1  Male

------------------------------------------------------------------------------------------------------------------------
bw                                                                                                           Birthweight
------------------------------------------------------------------------------------------------------------------------

                   type:  numeric (float)

                  range:  [946,4286]                units:  1
         unique values:  558                    missing .:  0/5,814

                   mean:   2943.94
              std. dev:   436.032

           percentiles:        10%       25%       50%       75%       90%
                              2406      2676      2964      3226      3480

------------------------------------------------------------------------------------------------------------------------
epdsw2                                                                                    Elapse weeks of 1.EPDS at 2W
------------------------------------------------------------------------------------------------------------------------

                   type:  numeric (float)

                  range:  [.71,4.71]                units:  .01
         unique values:  24                     missing .:  12/5,814

                   mean:   2.27746
              std. dev:   .372939

           percentiles:        10%       25%       50%       75%       90%
                              1.99      2.14      2.14      2.28      2.71

------------------------------------------------------------------------------------------------------------------------
epdsw4                                                                                    Elapse weeks of 2.EPDS at 4W
------------------------------------------------------------------------------------------------------------------------

                   type:  numeric (float)

                  range:  [2.71,11.57]              units:  .01
         unique values:  41                     missing .:  0/5,814
```

```
                mean:   4.42364
           std. dev:   .807883

        percentiles:        10%       25%       50%       75%       90%
                           3.99      4.14      4.14      4.42      4.99

--------------------------------------------------------------------------------------------------------------------------------
epdsw10                                                                                        Elapse weeks of 3.EPDS at 10W
--------------------------------------------------------------------------------------------------------------------------------

                type:  numeric (float)

               range:  [4.28,19.14]                 units:  .01
       unique values:  48                       missing .:  1,002/5,814

                mean:   8.59077
           std. dev:   1.06078

        percentiles:        10%       25%       50%       75%       90%
                           7.99      8.14      8.28      8.71      9.42

--------------------------------------------------------------------------------------------------------------------------------
faag01                                                                                           Father's age at birth (yrs)
--------------------------------------------------------------------------------------------------------------------------------

                type:  numeric (float)

               range:  [18.850103,53.431896]        units:  1.000e-06
       unique values:  896                      missing .:  0/5,814

                mean:   33.5507
           std. dev:   5.76045

        percentiles:        10%       25%       50%       75%       90%
                        26.5681   29.6783    33.295   37.0431   41.1444

--------------------------------------------------------------------------------------------------------------------------------
faed01                                                                                              Father's education (yrs)
--------------------------------------------------------------------------------------------------------------------------------

                type:  numeric (byte)

               range:  [9,26]                       units:  1
       unique values:  16                       missing .:  0/5,814
```

```
                 mean:   14.2508
             std. dev:   2.63563

          percentiles:        10%       25%       50%       75%       90%
                               12        12        14        16        18

------------------------------------------------------------------------------------------------------------------------
ga                                                                                             Gestational age at birth
------------------------------------------------------------------------------------------------------------------------

                 type:  numeric (float)

                range:  [30.139999,42.139999]        units:  1.000e-06
        unique values:  74                       missing .:  0/5,814

                 mean:   38.9673
             std. dev:   1.49481

          percentiles:        10%       25%       50%       75%       90%
                            37.28     38.14     39.14        40     40.71

------------------------------------------------------------------------------------------------------------------------
hbcm10                                                                                     Time elapsed 10M in Months
------------------------------------------------------------------------------------------------------------------------

                 type:  numeric (float)

                range:  [9.2,13.41]                  units:  .01
        unique values:  86                       missing .:  198/5,814

                 mean:   10.4529
             std. dev:   .485284

          percentiles:        10%       25%       50%       75%       90%
                            10.02     10.15     10.32     10.65     11.04

------------------------------------------------------------------------------------------------------------------------
hbcm14                                                                                     Time elapsed 14M in Months
------------------------------------------------------------------------------------------------------------------------

                 type:  numeric (float)

                range:  [12.13,20.64]                units:  .01
        unique values:  96                       missing .:  540/5,814

                 mean:   14.5114
```

```
          std. dev:   .640242

       percentiles:        10%       25%       50%       75%       90%
                         14.03     14.13     14.36     14.69     15.18

------------------------------------------------------------------------------------------------------------------------
hbcm18                                                                                  Time elapsed 18M in Month
------------------------------------------------------------------------------------------------------------------------

              type:  numeric (float)

             range:  [16.6,23.2]                  units:  .01
     unique values:  111                      missing .:  270/5,814

              mean:   18.6149
          std. dev:   .724087

       percentiles:        10%       25%       50%       75%       90%
                         18.01     18.17     18.44     18.86     19.46

------------------------------------------------------------------------------------------------------------------------
hbcm24                                                                                  Time elapsed 24M in Month
------------------------------------------------------------------------------------------------------------------------

              type:  numeric (float)

             range:  [21.03,33.82]                units:  .01
     unique values:  126                      missing .:  342/5,814

              mean:   24.7265
          std. dev:   .943389

       percentiles:        10%       25%       50%       75%       90%
                         24.03    24.205     24.49     24.95      25.7

------------------------------------------------------------------------------------------------------------------------
hbcm32                                                                                  Time elapsed 32M in Month
------------------------------------------------------------------------------------------------------------------------

              type:  numeric (float)

             range:  [28.79,59.63]                units:  .01
     unique values:  170                      missing .:  516/5,814

              mean:   33.2039
          std. dev:   1.96684
```

```
    percentiles:        10%       25%       50%       75%       90%
                      31.95     32.28     32.84     33.72     34.81

-------------------------------------------------------------------------------------------------------------------------------------
hbcm40                                                                                          Time elapsed 40M in Month
-------------------------------------------------------------------------------------------------------------------------------------

                  type:  numeric (float)

                 range:  [32.77,59.63]                units:  .01
         unique values:  200                      missing .:  510/5,814

                  mean:   39.8236
              std. dev:   2.17573

           percentiles:        10%       25%       50%       75%       90%
                             38.13     38.52     39.41     40.46     41.91

-------------------------------------------------------------------------------------------------------------------------------------
mlelt                                                                                                       (unlabeled)
-------------------------------------------------------------------------------------------------------------------------------------

                  type:  numeric (float)

                 range:  [20,80]                      units:  1.000e-06
         unique values:  1,795                    missing .:  571/5,814

                  mean:   49.0846
              std. dev:   9.99087

           percentiles:        10%       25%       50%       75%       90%
                           36.0387   42.6633   49.8449   55.9737   60.8195

-------------------------------------------------------------------------------------------------------------------------------------
moag01                                                                                      Mother's age at child's birth
-------------------------------------------------------------------------------------------------------------------------------------

                  type:  numeric (float)

                 range:  [17.735798,44.555782]        units:  1.000e-06
         unique values:  901                      missing .:  0/5,814

                  mean:   31.7459
              std. dev:   5.02203
```

```
     percentiles:        10%       25%       50%       75%       90%
                     25.1417   28.2081   31.8686   35.3895   38.2122

------------------------------------------------------------------------------------------------------------------------
moed01                                                                         Mother's educational history (yrs)
------------------------------------------------------------------------------------------------------------------------

                  type:  numeric (byte)

                 range:  [9,23]                       units:  1
         unique values:  14                       missing .:  0/5,814

                  mean:   13.9494
              std. dev:   1.91435

           percentiles:        10%       25%       50%       75%       90%
                                12        12        14        16        16

------------------------------------------------------------------------------------------------------------------------
mopd01                                                                                      Mother's EPDS at 2w
------------------------------------------------------------------------------------------------------------------------

                  type:  numeric (float)

                 range:  [0,22]                       units:  1
         unique values:  21                       missing .:  12/5,814

                  mean:    3.8635
              std. dev:   3.56301

           percentiles:        10%       25%       50%       75%       90%
                                 0         1         3         5         9

------------------------------------------------------------------------------------------------------------------------
mopd02                                                                                      Mother's EPDS at 4w
------------------------------------------------------------------------------------------------------------------------

                  type:  numeric (float)

                 range:  [0,21]                       units:  1
         unique values:  21                       missing .:  0/5,814

                  mean:   3.05986
              std. dev:   3.44116

           percentiles:        10%       25%       50%       75%       90%
```

```
                         0       1       2       4       7

-----------------------------------------------------------------------------------------------------------------
mopd3                                            PPD Early/Late 0/No PPD 1/Early PPD(2-4w) 2/Late PPD(5-12w)
-----------------------------------------------------------------------------------------------------------------

              type:  numeric (float)
             label:  labppd3

             range:  [0,2]                    units:  1
     unique values:  3                    missing .:  0/5,814

        tabulation:  Freq.   Numeric  Label
                     4,938         0  PPD-
                       618         1  PPD_Early
                       258         2  PPD_Late

-----------------------------------------------------------------------------------------------------------------
mopd03                                                                                  Mother's EPDS at 8w
-----------------------------------------------------------------------------------------------------------------

              type:  numeric (float)

             range:  [0,28]                   units:  1
     unique values:  21                   missing .:  1,002/5,814

              mean:   2.59476
          std. dev:   3.23768

       percentiles:        10%       25%       50%       75%       90%
                             0         0         1         4         7

-----------------------------------------------------------------------------------------------------------------
mopsyafdfor0                                     Maternal LIFETIME Affect Disord BY the index preg (29600-99)
-----------------------------------------------------------------------------------------------------------------

              type:  numeric (float)
             label:  labny

             range:  [0,1]                    units:  1
     unique values:  2                    missing .:  0/5,814

        tabulation:  Freq.   Numeric  Label
                     5,220         0  No
                       594         1  Yes
```

```
-------------------------------------------------------------------------------------------------------------------
mopsyafdfor40                                         Maternal LIFETIME Affect Disord during 0-40M (29600-99)
-------------------------------------------------------------------------------------------------------------------

              type:  numeric (float)
             label:  labny

             range:  [0,1]                    units:  1
     unique values:  2                    missing .:  0/5,814

        tabulation:  Freq.   Numeric  Label
                     4,914         0  No
                       900         1  Yes

-------------------------------------------------------------------------------------------------------------------
mopsyanxfor0                              Maternal LIFETIME Anxiety Disord BY the index preg (30000/30041:30928)
-------------------------------------------------------------------------------------------------------------------

              type:  numeric (float)
             label:  labny

             range:  [0,1]                    units:  1
     unique values:  2                    missing .:  0/5,814

        tabulation:  Freq.   Numeric  Label
                     5,604         0  No
                       210         1  Yes

-------------------------------------------------------------------------------------------------------------------
mopsyanxfor40                                 Maternal LIFETIME Anxiety Disord during 0-40M (30000/30041:30928)
-------------------------------------------------------------------------------------------------------------------

              type:  numeric (float)
             label:  labny

             range:  [0,1]                    units:  1
     unique values:  2                    missing .:  0/5,814

        tabulation:  Freq.   Numeric  Label
                     5,496         0  No
                       318         1  Yes

-------------------------------------------------------------------------------------------------------------------
parit3                                                         Number of children born before 0/0 1/1 2/2+
-------------------------------------------------------------------------------------------------------------------
```

```
              type:  numeric (float)
             label:  labparit3, but 2 nonmissing values are not labeled

             range:  [0,2]                        units:  1
     unique values:  3                        missing .:  0/5,814

        tabulation:  Freq.   Numeric  Label
                     2,910         0
                     2,178         1
                       726         2  2+

-----------------------------------------------------------------------------------------------------------------------
twin                                                                                   Twins/multiple births
-----------------------------------------------------------------------------------------------------------------------

              type:  numeric (float)

             range:  [0,1]                        units:  1
     unique values:  2                        missing .:  0/5,814

        tabulation:  Freq.  Value
                     5,634  0
                       180  1

-----------------------------------------------------------------------------------------------------------------------
bf                                                                          Duration of breastfeeding in month
-----------------------------------------------------------------------------------------------------------------------

              type:  numeric (float)

             range:  [0,23.07]                     units:  1.000e-08
     unique values:  272                       missing .:  0/5,814

              mean:   9.80104
         std. dev:    6.15872

       percentiles:        10%       25%       50%       75%       90%
                          1.05      4.37     10.22     14.43     18.37

-----------------------------------------------------------------------------------------------------------------------
bf3                                      Duration of breastfeeding in month categorized 0: bf=0, 1: 0<bf<6, 2: bf>=6
-----------------------------------------------------------------------------------------------------------------------

              type:  numeric (float)

             range:  [0,2]                        units:  1
```

```
       unique values:  3                    missing .:  0/5,814

          tabulation:  Freq.  Value
                         330  0
                       1,338  1
                       4,146  2

. log c
      name:  <unnamed>
       log:  C:¥Kjt¥__Text¥18097Paper_Peerj_Aoyagi¥181031codebook_long.log
  log type:  text
 closed on:  31 Oct 2018, 10:47:21
------------------------------------------------------------------------------------------------------------------------
```
