# Supplementary material for: Association of late-onset postpartum depression of mothers with expressive language development during infancy and early childhood: the HBC study
Source: PeerJ. 2019 Mar 6;7:e6566. doi: 10.7717/peerj.6566 (PMC6408909; doi:10.7717/peerj.6566)
Supplement: Supplemental Information 6 — All the codes used in the analyses, together with the outputs, are provided. The anaylses were conducted using Stata version 15.1. [file peerj-07-6566-s006.pdf]

```
  ___  ____  ____  ____  ____(R)
 /__    /   ____/   /   ____/
___/   /   /___/   /   /___/
  Statistics/Data Analysis
```

User: S Aoyagi
Project: HBC study

---

name: **<unnamed>**
log: **C:\stata_datafolder\20180528_HBC\peerJ_log_table1.smcl**
log type: **smcl**
opened on: **23 Oct 2018, 15:59:33**

```
. 
. * ======================
. * Table 1
. * ======================
. tab1 male parit3 twin bf3 mopsyafdfor0 mopsyanxfor0 mopsyafdfor40 mopsyanxfor40
```

-> tabulation of male

Gender of the child 0/female 1/male	Freq.	Percent	Cum.
Female	**478**	**49.33**	**49.33**
Male	**491**	**50.67**	**100.00**
Total	**969**	**100.00**	

-> tabulation of parit3

Number of children born before 0/0 1/1 2/2+	Freq.	Percent	Cum.
0	**485**	**50.05**	**50.05**
1	**363**	**37.46**	**87.51**
2+	**121**	**12.49**	**100.00**
Total	**969**	**100.00**	

-> tabulation of twin

Twins/multiple births	Freq.	Percent	Cum.
0	**939**	**96.90**	**96.90**
1	**30**	**3.10**	**100.00**
Total	**969**	**100.00**	

-> tabulation of bf3

RECODE of bf	Freq.	Percent	Cum.
0	**55**	**5.68**	**5.68**
1	**223**	**23.01**	**28.69**
2	**691**	**71.31**	**100.00**
Total	**969**	**100.00**	

-> tabulation of mopsyafdfor0

Maternal LIFETIME Affect Disord BY the index preg (29600-99)	Freq.	Percent	Cum.
No	**870**	**89.78**	**89.78**
Yes	**99**	**10.22**	**100.00**
Total	**969**	**100.00**	

-> tabulation of mopsyanxfor0

Maternal LIFETIME Anxiety Disord BY the index preg (30000/30041:30928)	Freq.	Percent	Cum.
No	**934**	**96.39**	**96.39**
Yes	**35**	**3.61**	**100.00**
Total	**969**	**100.00**	

-> tabulation of mopsyafdfor40

Maternal LIFETIME Affect Disord during 0-40M (29600-99)	Freq.	Percent	Cum.
No	**819**	**84.52**	**84.52**
Yes	**150**	**15.48**	**100.00**
Total	**969**	**100.00**	

-> tabulation of mopsyanxfor40

Maternal LIFETIME Anxiety Disord during 0-40M (30000/30041:30928)	Freq.	Percent	Cum.
No	**916**	**94.53**	**94.53**
Yes	**53**	**5.47**	**100.00**
Total	**969**	**100.00**	

6 . sum bw ga moag01 moed01 faag01 faed01 bf

Variable	Obs	Mean	Std. Dev.	Min	Max
bw	**969**	**2943.937**	**436.2201**	**946**	**4286**
ga	**969**	**38.9673**	**1.495451**	**30.14**	**42.14**
moag01	**969**	**31.74589**	**5.024193**	**17.7358**	**44.55578**
moed01	**969**	**13.94943**	**1.915175**	**9**	**23**
faag01	**969**	**33.55073**	**5.762924**	**18.8501**	**53.4319**
faed01	**969**	**14.25077**	**2.636768**	**9**	**26**
bf	**969**	**9.801042**	**6.161373**	**0**	**23.07**

```
 7 . tab mopd3

         PPD
  Early/Late
    0/No PPD
     1/Early
   PPD(2-4w)
      2/Late
  PPD(5-12w)        Freq.     Percent        Cum.
                                                
        PPD-          823       84.93       84.93
   PPD_Early          103       10.63       95.56
    PPD_Late           43        4.44      100.00
                                                
       Total          969      100.00

 8 . sum mlelt10 mlelt14 mlelt18 mlelt24 mlelt32 mlelt40

    Variable          Obs        Mean    Std. Dev.       Min        Max

     mlelt10          914    48.11256    9.687399   24.11217   78.67344
     mlelt14          849    49.14698     10.4845         20   70.43153
     mlelt18          898    48.58352    9.543554         20   76.60195
     mlelt24          874     49.4513    9.935041         20         80
     mlelt32          849    49.27621    9.940606         20         80

     mlelt40          859    50.01843    10.28115         20   78.53828

 9 .
10 . log c
      name:  <unnamed>
       log:  C:\stata_datafolder\20180528_HBC\peerJ_log_table1.smcl
  log type:  smcl
 closed on:  23 Oct 2018, 15:59:34
```

```
                                                      ___  ____  ____  ____  ____(R)
                                                     /__    /   ____/   /   ____/
                                                    ___/   /   /___/   /   /___/
                                                      Statistics/Data Analysis

                                                        User: S Aoyagi
                                                     Project: HBC study
-------------------------------------------------------------------------------------------
      name:  <unnamed>
       log:  C:\stata_datafolder\20180528_HBC\peerJ_log_table2.smcl
  log type:  smcl
 opened on:  23 Oct 2018, 15:59:34

1 .
2 . * =======================
3 . * Table 2
4 . * =======================
5 .
6 . *** Crude  ***
7 . mi est: reg mlelt10 i.mopd3

  Multiple-imputation estimates                  Imputations       =         20
  Linear regression                              Number of obs     =        969
                                                 Average RVI       =     0.0367
                                                 Largest FMI       =     0.0383
                                                 Complete DF       =        966
  DF adjustment:   Small sample                  DF:     min       =     866.18
                                                         avg       =     879.21
                                                         max       =     886.21
  Model F test:       Equal FMI                  F(   2,  927.7)   =       0.51
  Within VCE type:          OLS                  Prob > F          =     0.5977

  ------------------------------------------------------------------------------
       mlelt10 |      Coef.   Std. Err.      t    P>|t|     [95% Conf. Interval]
  -------------+----------------------------------------------------------------
         mopd3 |
     PPD_Early |   .9404046   1.031426     0.91   0.362    -1.083982    2.964792
      PPD_Late |  -.5758349   1.539434    -0.37   0.708    -3.597197    2.445527
               |
         _cons |   47.99015   .3430813   139.88   0.000      47.3168     48.6635
  ------------------------------------------------------------------------------

8 . mi est: reg mlelt14 i.mopd3

  Multiple-imputation estimates                  Imputations       =         20
  Linear regression                              Number of obs     =        969
                                                 Average RVI       =     0.0739
                                                 Largest FMI       =     0.0892
                                                 Complete DF       =        966
  DF adjustment:   Small sample                  DF:     min       =     646.25
                                                         avg       =     734.08
                                                         max       =     843.52
  Model F test:       Equal FMI                  F(   2,  843.9)   =       1.13
  Within VCE type:          OLS                  Prob > F          =     0.3221

  ------------------------------------------------------------------------------
       mlelt14 |      Coef.   Std. Err.      t    P>|t|     [95% Conf. Interval]
  -------------+----------------------------------------------------------------
         mopd3 |
     PPD_Early |  -.3457374   1.121873    -0.31   0.758    -2.547728    1.856253
      PPD_Late |  -2.539464    1.71959    -1.48   0.140    -5.916123     .837195
               |
         _cons |   49.23095   .3801116   129.52   0.000     48.48468    49.97722
  ------------------------------------------------------------------------------

9 . mi est: reg mlelt18 i.mopd3

  Multiple-imputation estimates                  Imputations       =         20
  Linear regression                              Number of obs     =        969
                                                 Average RVI       =     0.0685
                                                 Largest FMI       =     0.0976
                                                 Complete DF       =        966
  DF adjustment:   Small sample                  DF:     min       =     610.38
                                                         avg       =     758.83
                                                         max       =     882.80
  Model F test:       Equal FMI                  F(   2,  807.4)   =       3.93
  Within VCE type:          OLS                  Prob > F          =     0.0199
```

mlelt18	Coef.	Std. Err.	t	P>|t|	[95% Conf.	Interval]
mopd3						
PPD_Early	.7976991	1.048625	0.76	0.447	-1.261652	2.857051
PPD_Late	-4.092387	1.536894	-2.66	0.008	-7.109306	-1.075469
_cons	48.51407	.3381539	143.47	0.000	47.8504	49.17775

10 . mi est: reg mlelt24 i.mopd3

```
Multiple-imputation estimates                   Imputations       =         20
Linear regression                               Number of obs     =        969
                                                Average RVI       =     0.0925
                                                Largest FMI       =     0.0946
                                                Complete DF       =        966
DF adjustment:   Small sample                   DF:     min       =     623.05
                                                        avg       =     694.40
                                                        max       =     764.35
Model F test:       Equal FMI                   F(   2,  782.9)   =       7.40
Within VCE type:          OLS                   Prob > F          =     0.0007
```

mlelt24	Coef.	Std. Err.	t	P>|t|	[95% Conf.	Interval]
mopd3						
PPD_Early	.3299884	1.074909	0.31	0.759	-1.780466	2.440443
PPD_Late	-6.144574	1.623328	-3.79	0.000	-9.33243	-2.956717
_cons	49.49499	.355594	139.19	0.000	48.79694	50.19305

11 . mi est: reg mlelt32 i.mopd3

```
Multiple-imputation estimates                   Imputations       =         20
Linear regression                               Number of obs     =        969
                                                Average RVI       =     0.1320
                                                Largest FMI       =     0.1422
                                                Complete DF       =        966
DF adjustment:   Small sample                   DF:     min       =     446.31
                                                        avg       =     568.43
                                                        max       =     711.95
Model F test:       Equal FMI                   F(   2,  759.0)   =       5.03
Within VCE type:          OLS                   Prob > F          =     0.0068
```

mlelt32	Coef.	Std. Err.	t	P>|t|	[95% Conf.	Interval]
mopd3						
PPD_Early	-.0231768	1.084079	-0.02	0.983	-2.151551	2.105197
PPD_Late	-5.185783	1.65727	-3.13	0.002	-8.441174	-1.930391
_cons	49.18169	.375297	131.05	0.000	48.44412	49.91926

12 . mi est: reg mlelt40 i.mopd3

```
Multiple-imputation estimates                   Imputations       =         20
Linear regression                               Number of obs     =        969
                                                Average RVI       =     0.0642
                                                Largest FMI       =     0.0729
                                                Complete DF       =        966
DF adjustment:   Small sample                   DF:     min       =     718.07
                                                        avg       =     764.16
                                                        max       =     840.27
Model F test:       Equal FMI                   F(   2,  835.5)   =       6.07
Within VCE type:          OLS                   Prob > F          =     0.0024
```

```
------------------------------------------------------------------------------
     mlelt40 |      Coef.   Std. Err.      t    P>|t|     [95% Conf. Interval]
-------------+----------------------------------------------------------------
       mopd3 |
   PPD_Early |  -1.142563   1.117385    -1.02   0.307    -3.336296    1.051169
    PPD_Late |   -5.67262    1.66922    -3.40   0.001    -8.949634   -2.395606
             |
       _cons |   50.13217   .3672242   136.52   0.000     49.41138    50.85295
------------------------------------------------------------------------------

13 .
14 . ***  Model 1 ***
15 . mi est: reg mlelt10 i.mopd3 mopsyafdfor0 mopsyanxfor0

   Multiple-imputation estimates                   Imputations       =         20
   Linear regression                               Number of obs     =        969
                                                   Average RVI       =     0.0424
                                                   Largest FMI       =     0.0579
                                                   Complete DF       =        964
   DF adjustment:   Small sample                   DF:     min       =     783.22
                                                           avg       =     847.19
                                                           max       =     879.76
   Model F test:       Equal FMI                   F(   4,  938.6)   =       0.56
   Within VCE type:          OLS                   Prob > F          =     0.6928

------------------------------------------------------------------------------
     mlelt10 |      Coef.   Std. Err.      t    P>|t|     [95% Conf. Interval]
-------------+----------------------------------------------------------------
       mopd3 |
   PPD_Early |   1.098543    1.04847     1.05   0.295    -.9593552    3.156441
    PPD_Late |  -.5322518   1.543748    -0.34   0.730    -3.562111    2.497607
             |
mopsyafdfor0 |  -.6912348   1.081106    -0.64   0.523    -2.813136    1.430667
mopsyanxfor0 |  -1.326611   1.750182    -0.76   0.449    -4.762214    2.108993
       _cons |   48.08994   .3573221   134.58   0.000     47.38862    48.79127
------------------------------------------------------------------------------

16 . mi est: reg mlelt14 i.mopd3 mopsyafdfor0 mopsyanxfor0

   Multiple-imputation estimates                   Imputations       =         20
   Linear regression                               Number of obs     =        969
                                                   Average RVI       =     0.1108
                                                   Largest FMI       =     0.1929
                                                   Complete DF       =        964
   DF adjustment:   Small sample                   DF:     min       =     315.31
                                                           avg       =     628.71
                                                           max       =     803.94
   Model F test:       Equal FMI                   F(   4,  829.2)   =       1.94
   Within VCE type:          OLS                   Prob > F          =     0.1019

------------------------------------------------------------------------------
     mlelt14 |      Coef.   Std. Err.      t    P>|t|     [95% Conf. Interval]
-------------+----------------------------------------------------------------
       mopd3 |
   PPD_Early |  -.4194166   1.140395    -0.37   0.713     -2.65792    1.819087
    PPD_Late |  -2.695264   1.724285    -1.56   0.119    -6.081418    .6908895
             |
mopsyafdfor0 |   1.404217    1.27367     1.10   0.271    -1.101749    3.910182
mopsyanxfor0 |  -4.347498    1.92188    -2.26   0.024    -8.121271   -.5737255
       _cons |   49.25926   .3918392   125.71   0.000     48.49002    50.02849
------------------------------------------------------------------------------
```

17 . mi est: reg mlelt18 i.mopd3 mopsyafdfor0 mopsyanxfor0

```
Multiple-imputation estimates                   Imputations       =         20
Linear regression                               Number of obs     =        969
                                                Average RVI       =     0.0666
                                                Largest FMI       =     0.0938
                                                Complete DF       =        964
DF adjustment:   Small sample                   DF:     min       =     625.37
                                                        avg       =     761.32
                                                        max       =     860.92
Model F test:       Equal FMI                   F(   4,  900.6)   =       2.43
Within VCE type:          OLS                   Prob > F          =     0.0461
```

mlelt18	Coef.	Std. Err.	t	P>|t|	[95% Conf.	Interval]
mopd3						
PPD_Early	1.015108	1.061202	0.96	0.339	-1.068842	3.099058
PPD_Late	-4.014289	1.539145	-2.61	0.009	-7.035618	-.99296
mopsyafdfor0	-1.095511	1.065228	-1.03	0.304	-3.186258	.9952364
mopsyanxfor0	-1.157566	1.746271	-0.66	0.508	-4.586343	2.27121
_cons	48.64124	.3519225	138.22	0.000	47.95051	49.33196

18 . mi est: reg mlelt24 i.mopd3 mopsyafdfor0 mopsyanxfor0

```
Multiple-imputation estimates                   Imputations       =         20
Linear regression                               Number of obs     =        969
                                                Average RVI       =     0.0689
                                                Largest FMI       =     0.0954
                                                Complete DF       =        964
DF adjustment:   Small sample                   DF:     min       =     618.80
                                                        avg       =     773.56
                                                        max       =     904.60
Model F test:       Equal FMI                   F(   4,  915.7)   =       5.48
Within VCE type:          OLS                   Prob > F          =     0.0002
```

mlelt24	Coef.	Std. Err.	t	P>|t|	[95% Conf.	Interval]
mopd3						
PPD_Early	.7427424	1.086062	0.68	0.494	-1.389569	2.875054
PPD_Late	-6.009584	1.622652	-3.70	0.000	-9.196156	-2.823012
mopsyafdfor0	-1.973772	1.09371	-1.80	0.071	-4.120277	.1727325
mopsyanxfor0	-2.684357	1.765034	-1.52	0.129	-6.148656	.7799425
_cons	49.74374	.3671233	135.50	0.000	49.02308	50.4644

19 . mi est: reg mlelt32 i.mopd3 mopsyafdfor0 mopsyanxfor0

```
Multiple-imputation estimates                   Imputations       =         20
Linear regression                               Number of obs     =        969
                                                Average RVI       =     0.1021
                                                Largest FMI       =     0.1362
                                                Complete DF       =        964
DF adjustment:   Small sample                   DF:     min       =     465.29
                                                        avg       =     665.83
                                                        max       =     876.93
Model F test:       Equal FMI                   F(   4,  894.1)   =       6.30
Within VCE type:          OLS                   Prob > F          =     0.0001
```

mlelt32	Coef.	Std. Err.	t	P>|t|	[95% Conf.	Interval]
mopd3						
PPD_Early	**.5517589**	**1.09028**	**0.51**	**0.613**	**-1.588751**	**2.692269**
PPD_Late	**-5.027259**	**1.649829**	**-3.05**	**0.002**	**-8.268131**	**-1.786387**
mopsyafdfor0	**-2.513651**	**1.104683**	**-2.28**	**0.023**	**-4.681782**	**-.34552**
mopsyanxfor0	**-4.82049**	**1.803565**	**-2.67**	**0.008**	**-8.361305**	**-1.279675**
_cons	**49.54447**	**.3853658**	**128.56**	**0.000**	**48.78719**	**50.30174**

20 . mi est: reg mlelt40 i.mopd3 mopsyafdfor0 mopsyanxfor0

Multiple-imputation estimates — Imputations = **20**
Linear regression — Number of obs = **969**
Average RVI = **0.0674**
Largest FMI = **0.0756**
Complete DF = **964**
DF adjustment: **Small sample** — DF: min = **704.90**
avg = **762.15**
max = **842.36**
Model F test: **Equal FMI** — F(**4**, **901.8**) = **3.30**
Within VCE type: **OLS** — Prob > F = **0.0107**

mlelt40	Coef.	Std. Err.	t	P>|t|	[95% Conf.	Interval]
mopd3						
PPD_Early	**-1.071272**	**1.13045**	**-0.95**	**0.344**	**-3.290543**	**1.147999**
PPD_Late	**-5.688264**	**1.67439**	**-3.40**	**0.001**	**-8.975494**	**-2.401034**
mopsyafdfor0	**-.0297501**	**1.172788**	**-0.03**	**0.980**	**-2.332326**	**2.272826**
mopsyanxfor0	**-1.891433**	**1.848599**	**-1.02**	**0.307**	**-5.519833**	**1.736967**
_cons	**50.19664**	**.3831647**	**131.01**	**0.000**	**49.44451**	**50.94877**

21 . mi est, vartable dftable

Multiple-imputation estimates — Imputations = **20**
Linear regression

Variance information

	Imputation variance					Relative
	Within	Between	Total	RVI	FMI	efficiency
mopd3						
PPD_Early	**1.19213**	**.081704**	**1.27792**	**.071963**	**.067715**	**.996626**
PPD_Late	**2.60619**	**.187995**	**2.80358**	**.075741**	**.07104**	**.996461**
mopsyafdfor0	**1.27245**	**.098074**	**1.37543**	**.080929**	**.075571**	**.996236**
mopsyanxfor0	**3.2682**	**.142018**	**3.41732**	**.045627**	**.043922**	**.997809**
_cons	**.138885**	**.007553**	**.146815**	**.057099**	**.054419**	**.997286**

Multiple-imputation estimates — Imputations = **20**
Linear regression — Number of obs = **969**
Average RVI = **0.0674**
Largest FMI = **0.0756**
Complete DF = **964**
DF adjustment: **Small sample** — DF: min = **704.90**
avg = **762.15**
max = **842.36**
Model F test: **Equal FMI** — F(**4**, **901.8**) = **3.30**
Within VCE type: **OLS** — Prob > F = **0.0107**

mlelt40	Coef.	Std. Err.	t	P>|t|	DF	% Increase Std. Err.
mopd3						
PPD_Early	**-1.071272**	**1.13045**	**-0.95**	**0.344**	**739.9**	**3.54**
PPD_Late	**-5.688264**	**1.67439**	**-3.40**	**0.001**	**725.1**	**3.72**
mopsyafdfor0	**-.0297501**	**1.172788**	**-0.03**	**0.980**	**704.9**	**3.97**
mopsyanxfor0	**-1.891433**	**1.848599**	**-1.02**	**0.307**	**842.4**	**2.26**
_cons	**50.19664**	**.3831647**	**131.01**	**0.000**	**798.5**	**2.82**

22 .

23 . *** Model 2***

24 . mi est, saving(es): reg mlelt10 i.mopd3 male i.parit3 twin bw ga moag01 moed01 faag01 faed01

Multiple-imputation estimates		Imputations =	**20**
Linear regression		Number of obs =	**969**
		Average RVI =	**0.0496**
		Largest FMI =	**0.1032**
		Complete DF =	**953**
DF adjustment:	**Small sample**	DF: min =	**581.86**
		avg =	**801.92**
		max =	**893.74**
Model F test:	**Equal FMI**	F(**15**, **943.3**) =	**3.86**
Within VCE type:	**OLS**	Prob > F =	**0.0000**

mlelt10	Coef.	Std. Err.	t	P>|t|	[95% Conf.	Interval]
mopd3						
PPD_Early	**.8710345**	**1.041762**	**0.84**	**0.403**	**-1.173774**	**2.915843**
PPD_Late	**.1020284**	**1.528052**	**0.07**	**0.947**	**-2.897155**	**3.101212**
male	**-1.717191**	**.6307713**	**-2.72**	**0.007**	**-2.955178**	**-.4792033**
parit3						
1	**-.1495385**	**.702698**	**-0.21**	**0.832**	**-1.528897**	**1.22982**
2+	**-1.323557**	**1.042312**	**-1.27**	**0.205**	**-3.36952**	**.7224057**
twin	**-2.534914**	**1.896175**	**-1.34**	**0.182**	**-6.256433**	**1.186606**
bw	**.0026749**	**.0009165**	**2.92**	**0.004**	**.0008761**	**.0044736**
ga	**.5415636**	**.2787435**	**1.94**	**0.052**	**-.0057163**	**1.088844**
moag01	**.1694629**	**.0978121**	**1.73**	**0.084**	**-.0225494**	**.3614751**
moed01	**.2016158**	**.1853392**	**1.09**	**0.277**	**-.1621588**	**.5653905**
faag01	**-.1903871**	**.0825895**	**-2.31**	**0.021**	**-.3524945**	**-.0282796**
faed01	**-.1700985**	**.1358444**	**-1.25**	**0.211**	**-.4367096**	**.0965126**
mopsyafdfor0	**-.4246509**	**1.062539**	**-0.40**	**0.690**	**-2.510149**	**1.660847**
mopsyanxfor0	**-.9252147**	**1.722562**	**-0.54**	**0.591**	**-4.306646**	**2.456217**
bf	**.0490565**	**.0530119**	**0.93**	**0.355**	**-.0550356**	**.1531487**
_cons	**20.37495**	**10.32898**	**1.97**	**0.049**	**.0883216**	**40.66158**

25 . erase es.ster

26 . mi est, saving(es): reg mlelt14 i.mopd3 male i.parit3 twin bw ga moag01 moed01 faag01 faed01

Multiple-imputation estimates		Imputations =	**20**
Linear regression		Number of obs =	**969**
		Average RVI =	**0.1142**
		Largest FMI =	**0.2044**
		Complete DF =	**953**
DF adjustment:	**Small sample**	DF: min =	**291.28**
		avg =	**600.45**
		max =	**831.32**
Model F test:	**Equal FMI**	F(**15**, **916.2**) =	**4.77**
Within VCE type:	**OLS**	Prob > F =	**0.0000**

mlelt14	Coef.	Std. Err.	t	P>|t|	[95% Conf.	Interval]
mopd3						
PPD_Early	**-.3269686**	**1.12844**	**-0.29**	**0.772**	**-2.542179**	**1.888242**
PPD_Late	**-1.345869**	**1.6818**	**-0.80**	**0.424**	**-4.648182**	**1.956443**
male	**-2.298936**	**.7077087**	**-3.25**	**0.001**	**-3.689086**	**-.9087865**
parit3						
1	**.6949351**	**.751933**	**0.92**	**0.356**	**-.7809753**	**2.170846**
2+	**1.1288**	**1.161984**	**0.97**	**0.332**	**-1.153933**	**3.411533**
twin	**-7.493737**	**2.089373**	**-3.59**	**0.000**	**-11.59602**	**-3.391457**
bw	**.0028484**	**.0010058**	**2.83**	**0.005**	**.0008737**	**.0048232**
ga	**.2817263**	**.310073**	**0.91**	**0.364**	**-.3276334**	**.8910861**
moag01	**.0150412**	**.106916**	**0.14**	**0.888**	**-.1949157**	**.2249981**
moed01	**.2261869**	**.2039137**	**1.11**	**0.268**	**-.1742065**	**.6265802**
faag01	**-.148341**	**.0936987**	**-1.58**	**0.114**	**-.3324945**	**.0358124**
faed01	**-.2067498**	**.1479416**	**-1.40**	**0.163**	**-.4971507**	**.083651**
mopsyafdfor0	**1.529316**	**1.252345**	**1.22**	**0.223**	**-.9354762**	**3.994109**
mopsyanxfor0	**-4.154759**	**1.891308**	**-2.20**	**0.028**	**-7.8691**	**-.4404168**
bf	**.0741398**	**.0577437**	**1.28**	**0.200**	**-.0392812**	**.1875607**
_cons	**34.36563**	**11.32666**	**3.03**	**0.003**	**12.10618**	**56.62508**

```
27 . erase es.ster

28 . mi est, saving(es): reg mlelt18 i.mopd3 male i.parit3 twin bw ga moag01 moed01 faag01 faed01

Multiple-imputation estimates                     Imputations       =         20
Linear regression                                 Number of obs     =        969
                                                  Average RVI       =     0.0653
                                                  Largest FMI       =     0.1179
                                                  Complete DF       =        953
DF adjustment:   Small sample                     DF:     min       =     525.28
                                                          avg       =     755.79
                                                          max       =     866.27
Model F test:       Equal FMI                     F(  15,  937.9)   =       4.83
Within VCE type:          OLS                     Prob > F          =     0.0000
```

mlelt18	Coef.	Std. Err.	t	P>|t|	[95% Conf.	Interval]
mopd3						
PPD_Early	**1.050064**	**1.052021**	**1.00**	**0.319**	**-1.016064**	**3.116192**
PPD_Late	**-3.390613**	**1.522403**	**-2.23**	**0.026**	**-6.379423**	**-.4018026**
male	**-2.912179**	**.6234488**	**-4.67**	**0.000**	**-4.135902**	**-1.688455**
parit3						
1	**-.4692415**	**.685234**	**-0.68**	**0.494**	**-1.814204**	**.8757207**
2+	**-.1629384**	**1.020839**	**-0.16**	**0.873**	**-2.1667**	**1.840824**
twin	**-1.955533**	**1.949862**	**-1.00**	**0.316**	**-5.786019**	**1.874953**
bw	**.0034592**	**.0008992**	**3.85**	**0.000**	**.0016943**	**.0052241**
ga	**.1014611**	**.2705559**	**0.38**	**0.708**	**-.4296386**	**.6325608**
moag01	**.1058792**	**.0955564**	**1.11**	**0.268**	**-.081692**	**.2934504**
moed01	**.3460992**	**.1849333**	**1.87**	**0.062**	**-.016976**	**.7091744**
faag01	**-.1430489**	**.0820183**	**-1.74**	**0.082**	**-.3040663**	**.0179685**
faed01	**-.0394917**	**.1346678**	**-0.29**	**0.769**	**-.3038257**	**.2248423**
mopsyafdfor0	**-.8939198**	**1.045057**	**-0.86**	**0.393**	**-2.945169**	**1.157329**
mopsyanxfor0	**-.9541358**	**1.716072**	**-0.56**	**0.578**	**-4.323843**	**2.415571**
bf	**.0931718**	**.0510348**	**1.83**	**0.068**	**-.0070032**	**.1933468**
_cons	**32.43671**	**9.953751**	**3.26**	**0.001**	**12.89504**	**51.97839**

```
29 . erase es.ster

30 . mi est, saving(es): reg mlelt24 i.mopd3 male i.parit3 twin bw ga moag01 moed01 faag01 faed01

   Multiple-imputation estimates                   Imputations       =         20
   Linear regression                               Number of obs     =        969
                                                   Average RVI       =     0.0787
                                                   Largest FMI       =     0.1182
                                                   Complete DF       =        953
   DF adjustment:   Small sample                   DF:     min       =     524.19
                                                           avg       =     725.21
                                                           max       =     903.94
   Model F test:       Equal FMI                   F(  15,  934.0)   =       6.06
   Within VCE type:          OLS                   Prob > F          =     0.0000
```

mlelt24	Coef.	Std. Err.	t	P>|t|	[95% Conf.	Interval]
mopd3						
PPD_Early	.8109585	1.069142	0.76	0.448	-1.288161	2.910078
PPD_Late	-5.585423	1.584617	-3.52	0.000	-8.697082	-2.473763
male	-3.566372	.655825	-5.44	0.000	-4.854144	-2.278599
parit3						
1	-.6071734	.7196928	-0.84	0.399	-2.020247	.8059005
2+	-.746756	1.03856	-0.72	0.472	-2.785026	1.291514
twin	-.0494989	1.969531	-0.03	0.980	-3.91672	3.817722
bw	.0035209	.0009271	3.80	0.000	.0017013	.0053405
ga	-.0076503	.2792567	-0.03	0.978	-.5558517	.5405512
moag01	.0841679	.0995211	0.85	0.398	-.1112253	.2795611
moed01	.3799466	.1905802	1.99	0.047	.0057787	.7541145
faag01	-.1068536	.086834	-1.23	0.219	-.2774389	.0637318
faed01	.1358571	.1394786	0.97	0.330	-.1379447	.4096589
mopsyafdfor0	-1.812845	1.069024	-1.70	0.090	-3.910975	.2852847
mopsyanxfor0	-2.422254	1.73237	-1.40	0.162	-5.822703	.9781951
bf	.1022363	.054542	1.87	0.061	-.0049107	.2093834
_cons	34.42886	10.15466	3.39	0.001	14.49593	54.36179

```
31 . erase es.ster

32 . mi est, saving(es): reg mlelt32 i.mopd3 male i.parit3 twin bw ga moag01 moed01 faag01 faed01

   Multiple-imputation estimates                   Imputations       =         20
   Linear regression                               Number of obs     =        969
                                                   Average RVI       =     0.1182
                                                   Largest FMI       =     0.1964
                                                   Complete DF       =        953
   DF adjustment:   Small sample                   DF:     min       =     306.72
                                                           avg       =     572.72
                                                           max       =     849.42
   Model F test:       Equal FMI                   F(  15,  918.4)   =       7.10
   Within VCE type:          OLS                   Prob > F          =     0.0000
```

mlelt32	Coef.	Std. Err.	t	P>|t|	[95% Conf.	Interval]
mopd3						
PPD_Early	.4888278	1.067324	0.46	0.647	-1.606734	2.58439
PPD_Late	-4.93519	1.59766	-3.09	0.002	-8.073268	-1.797112
male	-3.003931	.648895	-4.63	0.000	-4.277868	-1.729995
parit3						
1	-1.339164	.7391503	-1.81	0.071	-2.791551	.1132217
2+	-2.119378	1.069735	-1.98	0.048	-4.219867	-.0188903
twin	1.564748	2.005608	0.78	0.436	-2.375361	5.504858
bw	.0021925	.000951	2.31	0.021	.0003252	.0040599
ga	.2311157	.2999949	0.77	0.442	-.3591298	.8213611
moag01	.0530502	.1001627	0.53	0.597	-.1436388	.2497392

```
      moed01 |   .5514402   .2006255     2.75   0.006     .1569255    .9459549
      faag01 |  -.0179381   .0862527    -0.21   0.835    -.1873589    .1514827
      faed01 |   .3630529   .1424136     2.55   0.011     .0833532    .6427526
mopsyafdfor0 |  -2.420992   1.071761    -2.26   0.024    -4.524601   -.3173817
mopsyanxfor0 |  -4.287772   1.767952    -2.43   0.016    -7.759559   -.8159847
          bf |   .0830893    .053658     1.55   0.122    -.0222812    .1884597
       _cons |   21.53501   10.98398     1.96   0.051    -.0784922    43.14851
------------------------------------------------------------------------------

33 . erase es.ster

34 . mi est, saving(es): reg mlelt40 i.mopd3 male i.parit3 twin bw ga moag01 moed01 faag01 faed01

Multiple-imputation estimates                   Imputations       =         20
Linear regression                               Number of obs     =        969
                                                Average RVI       =     0.0802
                                                Largest FMI       =     0.1030
                                                Complete DF       =        953
DF adjustment:   Small sample                   DF:     min       =     582.47
                                                        avg       =     709.70
                                                        max       =     804.21
Model F test:       Equal FMI                   F(  15,  932.1)   =       9.50
Within VCE type:          OLS                   Prob > F          =     0.0000

------------------------------------------------------------------------------
     mlelt40 |      Coef.   Std. Err.      t    P>|t|     [95% Conf. Interval]
-------------+----------------------------------------------------------------
       mopd3 |
   PPD_Early |  -1.214338   1.078871    -1.13   0.261    -3.332364    .9036883
    PPD_Late |   -5.76544   1.588495    -3.63   0.000    -8.883975   -2.646905
             |
        male |  -3.593282   .6580216    -5.46   0.000    -4.885093   -2.301472
             |
      parit3 |
          1  |  -1.893667   .7305039    -2.59   0.010    -3.328004   -.4593308
         2+  |  -3.436261   1.071496    -3.21   0.001     -5.53963   -1.332891
             |
        twin |   3.256418   2.018897     1.61   0.107    -.7087864    7.221623
          bw |    .002997   .0009584     3.13   0.002     .0011152    .0048787
          ga |   .2741446   .2836192     0.97   0.334    -.2826378    .8309269
      moag01 |   .1421478   .1004962     1.41   0.158    -.0551447    .3394402
      moed01 |   .6772575   .1963483     3.45   0.001     .2916222    1.062893
      faag01 |  -.0586156   .0862234    -0.68   0.497     -.227912    .1106808
      faed01 |   .3781399   .1423462     2.66   0.008     .0986755    .6576042
mopsyafdfor0 |   .1321001    1.11257     0.12   0.906    -2.052416    2.316616
mopsyanxfor0 |  -1.107819   1.758155    -0.63   0.529    -4.558934    2.343296
          bf |   .1334867   .0539869     2.47   0.014     .0274929    .2394805
       _cons |   14.83287   10.35255     1.43   0.152    -5.490361     35.1561
------------------------------------------------------------------------------

35 . erase es.ster

36 .
37 . log c
      name:  <unnamed>
       log:  C:\stata_datafolder\20180528_HBC\peerJ_log_table2.smcl
  log type:  smcl
 closed on:  23 Oct 2018, 16:00:06
--------------------------------------------------------------------------------
```

```
      ___  ____  ____  ____  ____(R)
     /__    /   ____/   /   ____/
    ___/   /   /___/   /   /___/
      Statistics/Data Analysis

        User: S Aoyagi
     Project: HBC study
```

```
      name:  <unnamed>
       log:  C:\stata_datafolder\20180528_HBC\peerJ_log_table3.smcl
  log type:  smcl
 opened on:  23 Oct 2018, 16:00:09

1 .
2 . * ======================
3 . * Table 3
4 . * ======================
5 . mixed mlelt c.t##i.mopd3 male i.parit3 twin bw ga ///
  > moag01 moed01 faag01 faed01 mopsyafdfor0 mopsyanxfor0 c.bf ///
  > if t==10 | t==14 | t==18 | t==24 | t==32 | t==40 || id: t, cov(uns) vce(cluster moid) var

Performing EM optimization:

Performing gradient-based optimization:

Iteration 0:   log pseudolikelihood = -18680.201
Iteration 1:   log pseudolikelihood = -18680.199
Iteration 2:   log pseudolikelihood = -18680.199

Computing standard errors:

Mixed-effects regression                        Number of obs     =      5,243
Group variable: id                              Number of groups  =        969

                                                Obs per group:
                                                              min =          1
                                                              avg =        5.4
                                                              max =          6

                                                Wald chi2(18)     =     164.83
Log pseudolikelihood = -18680.199               Prob > chi2       =     0.0000

                                 (Std. Err. adjusted for 880 clusters in moid)
```

mlelt	Coef.	Robust Std. Err.	z	P>|z|	[95% Conf.	Interval]
t	.0593437	.0156311	3.80	0.000	.0287072	.0899801
mopd3						
PPD_Early	1.623658	1.138981	1.43	0.154	-.6087032	3.85602
PPD_Late	.8579228	1.827198	0.47	0.639	-2.723319	4.439164
mopd3#c.t						
PPD_Early	-.0576985	.0393408	-1.47	0.142	-.1348049	.019408
PPD_Late	-.1872499	.0615015	-3.04	0.002	-.3077907	-.0667092
male	-2.730121	.4413328	-6.19	0.000	-3.595117	-1.865124
parit3						
1	-.4705155	.4952003	-0.95	0.342	-1.44109	.5000592
2+	-.7684406	.6768341	-1.14	0.256	-2.095011	.5581298
twin	-1.773174	1.518988	-1.17	0.243	-4.750335	1.203987
bw	.0030259	.0006434	4.70	0.000	.0017649	.004287
ga	.2471392	.1930158	1.28	0.200	-.1311649	.6254434
moag01	.1048442	.0720179	1.46	0.145	-.0363083	.2459967
moed01	.351887	.1219185	2.89	0.004	.1129311	.5908428
faag01	-.1322044	.0598779	-2.21	0.027	-.249563	-.0148459
faed01	.0334222	.0977545	0.34	0.732	-.1581731	.2250175
mopsyafdfor0	-.5841811	.821888	-0.71	0.477	-2.195052	1.02669
mopsyanxfor0	-2.303599	1.301228	-1.77	0.077	-4.85396	.2467616
bf	.0837677	.0359467	2.33	0.020	.0133135	.1542219
_cons	25.90626	7.19946	3.60	0.000	11.79558	40.01695

Random-effects Parameters	Estimate	Robust Std. Err.	[95% Conf.	Interval]
**id**: Unstructured				
var(t)	.0896359	.0089564	.0736936	.1090269
var(_cons)	67.34337	5.721422	57.01349	79.54484
cov(t,_cons)	-1.722532	.2061632	-2.126604	-1.31846
var(Residual)	47.69364	1.405268	45.01739	50.529

```
6 .
7 . mixed mlelt c.t##c.t##i.mopd3 male i.parit3 twin bw ga ///
> moag01 moed01 faag01 faed01 mopsyafdfor0 mopsyanxfor0 c.bf ///
> if t==10 | t==14 | t==18 | t==24 | t==32 | t==40 || id: t, cov(uns) vce(cluster moid) var

Performing EM optimization:

Performing gradient-based optimization:

Iteration 0:   log pseudolikelihood = -18677.454
Iteration 1:   log pseudolikelihood = -18677.452
Iteration 2:   log pseudolikelihood = -18677.452

Computing standard errors:

Mixed-effects regression                        Number of obs     =      5,243
Group variable: id                              Number of groups  =        969

                                                Obs per group:
                                                              min =          1
                                                              avg =        5.4
                                                              max =          6

                                                Wald chi2(21)     =     167.57
Log pseudolikelihood = -18677.452               Prob > chi2       =     0.0000

                                 (Std. Err. adjusted for 880 clusters in moid)
```

mlelt	Coef.	Robust Std. Err.	z	P>|z|	[95% Conf.	Interval]
t	.0816206	.0707587	1.15	0.249	-.057064	.2203052
c.t#c.t	-.0004477	.0013209	-0.34	0.735	-.0030366	.0021413
mopd3						
PPD_Early	-.064274	2.239971	-0.03	0.977	-4.454536	4.325988
PPD_Late	6.469596	3.791699	1.71	0.088	-.9619975	13.90119
mopd3#c.t						
PPD_Early	.1087433	.1977273	0.55	0.582	-.278795	.4962816
PPD_Late	-.7392742	.3509749	-2.11	0.035	-1.427172	-.051376
mopd3#c.t#c.t						
PPD_Early	-.0033495	.0038155	-0.88	0.380	-.0108278	.0041289
PPD_Late	.0110837	.0068995	1.61	0.108	-.0024392	.0246066
male	-2.72596	.4409966	-6.18	0.000	-3.590298	-1.861623
parit3						
1	-.4762288	.4948588	-0.96	0.336	-1.446134	.4936767
2+	-.772158	.6768952	-1.14	0.254	-2.098848	.5545322
twin	-1.794797	1.498476	-1.20	0.231	-4.731757	1.142163
bw	.0030212	.0006434	4.70	0.000	.0017601	.0042822
ga	.2469372	.1928822	1.28	0.200	-.1311049	.6249794
moag01	.1058037	.0719801	1.47	0.142	-.0352746	.2468821
moed01	.3516084	.1217818	2.89	0.004	.1129205	.5902964
faag01	-.1323488	.0598678	-2.21	0.027	-.2496875	-.01501
faed01	.0331753	.0977071	0.34	0.734	-.1583272	.2246777
mopsyafdfor0	-.5891313	.8224517	-0.72	0.474	-2.201107	1.022844

mopsyanxfor0	-2.290827	1.300984	-1.76	0.078	-4.840709	.2590554
bf	.0838677	.0359452	2.33	0.020	.0134164	.1543189
_cons	25.6825	7.236295	3.55	0.000	11.49962	39.86538

Random-effects Parameters	Estimate	Robust Std. Err.	[95% Conf.	Interval]
**id**: Unstructured				
var(t)	.0896922	.0089459	.0737659	.1090571
var(_cons)	67.29316	5.707306	56.98733	79.46273
cov(t,_cons)	-1.722065	.2057981	-2.125422	-1.318708
var(Residual)	47.6264	1.39878	44.96226	50.4484

```
8 .
9 . log c
      name:  <unnamed>
       log:  C:\stata_datafolder\20180528_HBC\peerJ_log_table3.smcl
  log type:  smcl
 closed on:  23 Oct 2018, 16:00:30
```

```
  ___  ____  ____  ____  ____(R)
 /__    /   ____/   /   ____/
___/   /   /___/   /   /___/
  Statistics/Data Analysis
```

User: S Aoyagi
Project: HBC study

---

name: **<unnamed>**
log: **C:\stata_datafolder\20180528_HBC\peerJ_log_supplemental_table1.smcl**
log type: **smcl**
opened on: **23 Oct 2018, 15:59:34**

1 .
2 . * ======================
3 . * Suppelemental Table 1
4 . * ======================
5 . tab mopd3

PPD Early/Late 0/No PPD 1/Early PPD(2-4w) 2/Late PPD(5-12w)	Freq.	Percent	Cum.
PPD-	**823**	**84.93**	**84.93**
PPD_Early	**103**	**10.63**	**95.56**
PPD_Late	**43**	**4.44**	**100.00**
Total	**969**	**100.00**	

6 . tab male mopd3, col

Key
frequency<br>*column percentage*

Gender of the child 0/female 1/male	PPD Early/Late 0/No PPD 1/Early PPD(2-4w) 2/Late PPD(5-12w)			
	PPD-	PPD_Early	PPD_Late	Total
Female	**403**<br>**48.97**	**56**<br>**54.37**	**19**<br>**44.19**	**478**<br>**49.33**
Male	**420**<br>**51.03**	**47**<br>**45.63**	**24**<br>**55.81**	**491**<br>**50.67**
Total	**823**<br>**100.00**	**103**<br>**100.00**	**43**<br>**100.00**	**969**<br>**100.00**

7 . tab parit3 mopd3, col

Key
frequency<br>*column percentage*

Number of children born before 0/0 1/1 2/2+	PPD Early/Late 0/No PPD 1/Early PPD(2-4w) 2/Late PPD(5-12w)			
	PPD-	PPD_Early	PPD_Late	Total
0	**394**<br>**47.87**	**69**<br>**66.99**	**22**<br>**51.16**	**485**<br>**50.05**
1	**318**<br>**38.64**	**29**<br>**28.16**	**16**<br>**37.21**	**363**<br>**37.46**
2+	**111**<br>**13.49**	**5**<br>**4.85**	**5**<br>**11.63**	**121**<br>**12.49**

Total	**823**<br>**100.00**	**103**<br>**100.00**	**43**<br>**100.00**	**969**<br>**100.00**

8 . tab twin mopd3, col

Key
frequency<br>*column percentage*

Twins/multiple births	PPD Early/Late 0/No PPD 1/Early PPD(2-4w) 2/Late PPD(5-12w) PPD-	PPD_Early	PPD_Late	Total
0	**801**<br>**97.33**	**101**<br>**98.06**	**37**<br>**86.05**	**939**<br>**96.90**
1	**22**<br>**2.67**	**2**<br>**1.94**	**6**<br>**13.95**	**30**<br>**3.10**
Total	**823**<br>**100.00**	**103**<br>**100.00**	**43**<br>**100.00**	**969**<br>**100.00**

9 . tab bf3 mopd3, col

Key
frequency<br>*column percentage*

RECODE of bf	PPD Early/Late 0/No PPD 1/Early PPD(2-4w) 2/Late PPD(5-12w) PPD-	PPD_Early	PPD_Late	Total
0	**41**<br>**4.98**	**10**<br>**9.71**	**4**<br>**9.30**	**55**<br>**5.68**
1	**188**<br>**22.84**	**28**<br>**27.18**	**7**<br>**16.28**	**223**<br>**23.01**
2	**594**<br>**72.17**	**65**<br>**63.11**	**32**<br>**74.42**	**691**<br>**71.31**
Total	**823**<br>**100.00**	**103**<br>**100.00**	**43**<br>**100.00**	**969**<br>**100.00**

10 . tab mopsyafdfor0 mopd3, col

Key
frequency<br>*column percentage*

Maternal LIFETIME Affect Disord BY the index preg (29600-99)	PPD Early/Late 0/No PPD 1/Early PPD(2-4w) 2/Late PPD(5-12w) PPD-	PPD_Early	PPD_Late	Total
No	**756**<br>**91.86**	**78**<br>**75.73**	**36**<br>**83.72**	**870**<br>**89.78**
Yes	**67**<br>**8.14**	**25**<br>**24.27**	**7**<br>**16.28**	**99**<br>**10.22**

Total	**823**<br>**100.00**	**103**<br>**100.00**	**43**<br>**100.00**	**969**<br>**100.00**

11 . tab mopsyanxfor0 mopd3, col

Key
frequency<br>*column percentage*

Maternal LIFETIME Anxiety Disord BY the index preg (30000/300 41:30928)	PPD Early/Late 0/No PPD 1/Early PPD(2-4w) 2/Late PPD(5-12w) PPD-	PPD_Early	PPD_Late	Total
No	**796**<br>**96.72**	**96**<br>**93.20**	**42**<br>**97.67**	**934**<br>**96.39**
Yes	**27**<br>**3.28**	**7**<br>**6.80**	**1**<br>**2.33**	**35**<br>**3.61**
Total	**823**<br>**100.00**	**103**<br>**100.00**	**43**<br>**100.00**	**969**<br>**100.00**

12 . tab mopsyafdfor40 mopd3, col

Key
frequency<br>*column percentage*

Maternal LIFETIME Affect Disord during 0-40M (29600-99)	PPD Early/Late 0/No PPD 1/Early PPD(2-4w) 2/Late PPD(5-12w) PPD-	PPD_Early	PPD_Late	Total
No	**724**<br>**87.97**	**63**<br>**61.17**	**32**<br>**74.42**	**819**<br>**84.52**
Yes	**99**<br>**12.03**	**40**<br>**38.83**	**11**<br>**25.58**	**150**<br>**15.48**
Total	**823**<br>**100.00**	**103**<br>**100.00**	**43**<br>**100.00**	**969**<br>**100.00**

13 . tab mopsyanxfor40 mopd3, col

Key
frequency<br>*column percentage*

Maternal LIFETIME Anxiety Disord during 0-40M (30000/300 41:30928)	PPD Early/Late 0/No PPD 1/Early PPD(2-4w) 2/Late PPD(5-12w) PPD-	PPD_Early	PPD_Late	Total
No	**784**<br>**95.26**	**92**<br>**89.32**	**40**<br>**93.02**	**916**<br>**94.53**
Yes	**39**<br>**4.74**	**11**<br>**10.68**	**3**<br>**6.98**	**53**<br>**5.47**
Total	**823**<br>**100.00**	**103**<br>**100.00**	**43**<br>**100.00**	**969**<br>**100.00**

```
14 .
15 . codebook mopd3
```

mopd3

```
                 type:  numeric (float)
                label:  labppd3

                range:  [0,2]                        units:  1
        unique values:  3                        missing .:  0/969

           tabulation:  Freq.   Numeric  Label
                          823         0  PPD-
                          103         1  PPD_Early
                           43         2  PPD_Late
```

```
16 . sum bw if mopd3 ==0
```

Variable	Obs	Mean	Std. Dev.	Min	Max
bw	**823**	**2955.039**	**438.0591**	**946**	**4286**

```
17 . sum bw if mopd3 ==1
```

Variable	Obs	Mean	Std. Dev.	Min	Max
bw	**103**	**2881.883**	**427.2487**	**1448**	**3870**

```
18 . sum bw if mopd3 ==2
```

Variable	Obs	Mean	Std. Dev.	Min	Max
bw	**43**	**2880.093**	**413.1134**	**1808**	**3544**

```
19 .
20 . sum ga if mopd3 ==0
```

Variable	Obs	Mean	Std. Dev.	Min	Max
ga	**823**	**38.97111**	**1.513979**	**30.14**	**42.14**

21 . sum ga if mopd3 ==1

Variable	Obs	Mean	Std. Dev.	Min	Max
ga	**103**	**39.05102**	**1.364484**	**35.28**	**41.28**

22 . sum ga if mopd3 ==2

Variable	Obs	Mean	Std. Dev.	Min	Max
ga	**43**	**38.69372**	**1.437773**	**35**	**41.14**

23 .
24 . sum moag01 if mopd3 ==0

Variable	Obs	Mean	Std. Dev.	Min	Max
moag01	**823**	**31.72443**	**4.999849**	**17.7358**	**44.55578**

25 . sum moag01 if mopd3 ==1

Variable	Obs	Mean	Std. Dev.	Min	Max
moag01	**103**	**31.51378**	**4.996454**	**20.0219**	**41.18823**

26 . sum moag01 if mopd3 ==2

Variable	Obs	Mean	Std. Dev.	Min	Max
moag01	**43**	**32.71261**	**5.549553**	**20.72553**	**40.68446**

27 .
28 . sum moed01 if mopd3 ==0

Variable	Obs	Mean	Std. Dev.	Min	Max
moed01	**823**	**13.9514**	**1.911056**	**9**	**22**

29 . sum moed01 if mopd3 ==1

Variable	Obs	Mean	Std. Dev.	Min	Max
moed01	**103**	**13.83495**	**1.980776**	**9**	**23**

30 . sum moed01 if mopd3 ==2

Variable	Obs	Mean	Std. Dev.	Min	Max
moed01	**43**	**14.18605**	**1.854927**	**12**	**18**

31 .
32 . sum faag01 if mopd3 ==0

Variable	Obs	Mean	Std. Dev.	Min	Max
faag01	**823**	**33.5928**	**5.708739**	**19.6167**	**53.4319**

33 . sum faag01 if mopd3 ==1

Variable	Obs	Mean	Std. Dev.	Min	Max
faag01	**103**	**32.82113**	**5.658045**	**18.8501**	**52.32033**

34 . sum faag01 if mopd3 ==2

Variable	Obs	Mean	Std. Dev.	Min	Max
faag01	43	34.49335	6.900537	20.51472	48.32581

35 .
36 . sum faed01 if mopd3 ==0

Variable	Obs	Mean	Std. Dev.	Min	Max
faed01	823	14.29648	2.677213	9	26

37 . sum faed01 if mopd3 ==1

Variable	Obs	Mean	Std. Dev.	Min	Max
faed01	103	13.94175	2.36322	9	23

38 . sum faed01 if mopd3 ==2

Variable	Obs	Mean	Std. Dev.	Min	Max
faed01	43	14.11628	2.470871	9	22

39 .
40 . sum bf if mopd3 ==0

Variable	Obs	Mean	Std. Dev.	Min	Max
bf	823	9.976027	6.136888	0	22.15

41 . sum bf if mopd3 ==1

Variable	Obs	Mean	Std. Dev.	Min	Max
bf	103	8.620097	6.3209	0	23.07

42 . sum bf if mopd3 ==2

Variable	Obs	Mean	Std. Dev.	Min	Max
bf	43	9.280698	6.04404	0	18.63

43 .
44 . sum mlelt10 if mopd3 ==0

Variable	Obs	Mean	Std. Dev.	Min	Max
mlelt10	774	48.01591	9.627509	25.22819	78.67344

45 . sum mlelt10 if mopd3 ==1

Variable	Obs	Mean	Std. Dev.	Min	Max
mlelt10	99	49.11174	9.910802	26.43362	77.44672

46 . sum mlelt10 if mopd3 ==2

Variable	Obs	Mean	Std. Dev.	Min	Max
mlelt10	41	47.52445	10.35178	24.11217	66.19521

47 .
48 . sum mlelt14 if mopd3 ==0

Variable	Obs	Mean	Std. Dev.	Min	Max
mlelt14	**718**	**49.25063**	**10.53127**	**20**	**70.43153**

49 . sum mlelt14 if mopd3 ==1

Variable	Obs	Mean	Std. Dev.	Min	Max
mlelt14	**94**	**49.08838**	**10.1407**	**23.33756**	**66.41338**

50 . sum mlelt14 if mopd3 ==2

Variable	Obs	Mean	Std. Dev.	Min	Max
mlelt14	**37**	**47.28451**	**10.53487**	**20**	**64.74017**

51 .
52 . sum mlelt18 if mopd3 ==0

Variable	Obs	Mean	Std. Dev.	Min	Max
mlelt18	**770**	**48.69992**	**9.358142**	**20**	**76.60195**

53 . sum mlelt18 if mopd3 ==1

Variable	Obs	Mean	Std. Dev.	Min	Max
mlelt18	**91**	**49.44902**	**9.691576**	**20**	**68.54506**

54 . sum mlelt18 if mopd3 ==2

Variable	Obs	Mean	Std. Dev.	Min	Max
mlelt18	**37**	**44.03257**	**11.84964**	**20**	**63.92603**

55 .
56 . sum mlelt24 if mopd3 ==0

Variable	Obs	Mean	Std. Dev.	Min	Max
mlelt24	**743**	**49.69395**	**9.817472**	**20**	**78.44915**

57 . sum mlelt24 if mopd3 ==1

Variable	Obs	Mean	Std. Dev.	Min	Max
mlelt24	**93**	**50.22527**	**9.843545**	**22.62097**	**80**

58 . sum mlelt24 if mopd3 ==2

Variable	Obs	Mean	Std. Dev.	Min	Max
mlelt24	**38**	**42.81276**	**10.35191**	**20**	**57.73434**

59 .
60 . sum mlelt32 if mopd3 ==0

Variable	Obs	Mean	Std. Dev.	Min	Max
mlelt32	**725**	**49.51587**	**9.952311**	**20**	**80**

61 . sum mlelt32 if mopd3 ==1

Variable	Obs	Mean	Std. Dev.	Min	Max
mlelt32	**87**	**49.62434**	**8.892301**	**27.24406**	**70.68181**

62 . sum mlelt32 if mopd3 ==2

Variable	Obs	Mean	Std. Dev.	Min	Max
mlelt32	**37**	**43.76154**	**10.6808**	**20**	**67.57412**

63 .
64 . sum mlelt40 if mopd3 ==0

Variable	Obs	Mean	Std. Dev.	Min	Max
mlelt40	**730**	**50.47839**	**10.15976**	**20**	**78.53828**

65 . sum mlelt40 if mopd3 ==1

Variable	Obs	Mean	Std. Dev.	Min	Max
mlelt40	**91**	**49.01259**	**10.06591**	**20**	**77.74355**

66 . sum mlelt40 if mopd3 ==2

Variable	Obs	Mean	Std. Dev.	Min	Max
mlelt40	**38**	**43.59106**	**11.03924**	**20**	**65.74768**

67 .
68 . sum hbcm10 if mopd3 ==0

Variable	Obs	Mean	Std. Dev.	Min	Max
hbcm10	**793**	**10.43852**	**.4732154**	**9.2**	**13.41**

69 . sum hbcm10 if mopd3 ==1

Variable	Obs	Mean	Std. Dev.	Min	Max
hbcm10	**102**	**10.49029**	**.4696353**	**9.82**	**11.93**

70 . sum hbcm10 if mopd3 ==2

Variable	Obs	Mean	Std. Dev.	Min	Max
hbcm10	**41**	**10.63732**	**.6902428**	**9.96**	**13.11**

71 .
72 . sum hbcm14 if mopd3 ==0

Variable	Obs	Mean	Std. Dev.	Min	Max
hbcm14	**746**	**14.50909**	**.6474789**	**12.13**	**20.64**

73 . sum hbcm14 if mopd3 ==1

Variable	Obs	Mean	Std. Dev.	Min	Max
hbcm14	**95**	**14.46737**	**.5728321**	**13.28**	**17.58**

74 . sum hbcm14 if mopd3 ==2

Variable	Obs	Mean	Std. Dev.	Min	Max
hbcm14	**38**	**14.66737**	**.6567598**	**13.87**	**16.37**

75 .
76 . sum hbcm18 if mopd3 ==0

Variable	Obs	Mean	Std. Dev.	Min	Max
hbcm18	**789**	**18.59749**	**.7118261**	**16.6**	**23.2**

77 . sum hbcm18 if mopd3 ==1

Variable	Obs	Mean	Std. Dev.	Min	Max
hbcm18	**97**	**18.76392**	**.8572504**	**17.48**	**23.07**

78 . sum hbcm18 if mopd3 ==2

Variable	Obs	Mean	Std. Dev.	Min	Max
hbcm18	**38**	**18.59658**	**.5727426**	**17.78**	**20.54**

79 .
80 . sum hbcm24 if mopd3 ==0

Variable	Obs	Mean	Std. Dev.	Min	Max
hbcm24	**777**	**24.6999**	**.9156065**	**21.03**	**33.82**

81 . sum hbcm24 if mopd3 ==1

Variable	Obs	Mean	Std. Dev.	Min	Max
hbcm24	**95**	**24.83832**	**1.062347**	**22.74**	**29.52**

82 . sum hbcm24 if mopd3 ==2

Variable	Obs	Mean	Std. Dev.	Min	Max
hbcm24	**40**	**24.977**	**1.139431**	**23.53**	**29.52**

83 .
84 . sum hbcm32 if mopd3 ==0

Variable	Obs	Mean	Std. Dev.	Min	Max
hbcm32	**755**	**33.1773**	**2.018118**	**28.79**	**59.63**

85 . sum hbcm32 if mopd3 ==1

Variable	Obs	Mean	Std. Dev.	Min	Max
hbcm32	**89**	**33.45315**	**1.729674**	**31.16**	**43.88**

86 . sum hbcm32 if mopd3 ==2

Variable	Obs	Mean	Std. Dev.	Min	Max
hbcm32	**39**	**33.14923**	**1.403524**	**29.52**	**36.32**

```
 87 .
 88 . sum hbcm40 if mopd3 ==0

    Variable |        Obs        Mean    Std. Dev.       Min        Max
-------------+---------------------------------------------------------
      hbcm40 |        753    39.78726    2.196944      32.77      59.63

 89 . sum hbcm40 if mopd3 ==1

    Variable |        Obs        Mean    Std. Dev.       Min        Max
-------------+---------------------------------------------------------
      hbcm40 |         92    40.21652    2.306889      37.01      54.56

 90 . sum hbcm40 if mopd3 ==2

    Variable |        Obs        Mean    Std. Dev.       Min        Max
-------------+---------------------------------------------------------
      hbcm40 |         39    39.59795    1.179592      35.33      42.63

 91 .
 92 . sum epdsw2 if mopd3 ==0

    Variable |        Obs        Mean    Std. Dev.       Min        Max
-------------+---------------------------------------------------------
      epdsw2 |        821    2.266882    .3640507        .71       4.71

 93 . sum epdsw2 if mopd3 ==1

    Variable |        Obs        Mean    Std. Dev.       Min        Max
-------------+---------------------------------------------------------
      epdsw2 |        103    2.329806    .4295551        .85       3.71

 94 . sum epdsw2 if mopd3 ==2

    Variable |        Obs        Mean    Std. Dev.       Min        Max
-------------+---------------------------------------------------------
      epdsw2 |         43    2.353954    .3887473       1.71       3.42

 95 .
 96 . sum epdsw4 if mopd3 ==0

    Variable |        Obs        Mean    Std. Dev.       Min        Max
-------------+---------------------------------------------------------
      epdsw4 |        823     4.43339    .8219726       2.71      11.57

 97 . sum epdsw4 if mopd3 ==1

    Variable |        Obs        Mean    Std. Dev.       Min        Max
-------------+---------------------------------------------------------
      epdsw4 |        103     4.30699    .3994699       3.42       5.71

 98 . sum epdsw4 if mopd3 ==2

    Variable |        Obs        Mean    Std. Dev.       Min        Max
-------------+---------------------------------------------------------
      epdsw4 |         43    4.516512     1.18352       3.28       8.85

 99 .
100 . sum epdsw10 if mopd3 ==0

    Variable |        Obs        Mean    Std. Dev.       Min        Max
-------------+---------------------------------------------------------
     epdsw10 |        678    8.600959     1.10712       4.28      19.14
```

101 . sum epdsw10 if mopd3 ==1

Variable	Obs	Mean	Std. Dev.	Min	Max
epdsw10	**85**	**8.522588**	**.8013086**	**6.14**	**11.42**

102 . sum epdsw10 if mopd3 ==2

Variable	Obs	Mean	Std. Dev.	Min	Max
epdsw10	**39**	**8.562308**	**.6886503**	**7.42**	**11.28**

103 .
104 . sum mopd01 if mopd3 ==0

Variable	Obs	Mean	Std. Dev.	Min	Max
mopd01	**821**	**2.783191**	**2.162149**	**0**	**8**

105 . sum mopd01 if mopd3 ==1

Variable	Obs	Mean	Std. Dev.	Min	Max
mopd01	**103**	**11.68932**	**3.013138**	**5**	**22**

106 . sum mopd01 if mopd3 ==2

Variable	Obs	Mean	Std. Dev.	Min	Max
mopd01	**43**	**5.744186**	**1.513322**	**2**	**8**

107 .
108 . sum mopd02 if mopd3 ==0

Variable	Obs	Mean	Std. Dev.	Min	Max
mopd02	**823**	**2.153098**	**2.085376**	**0**	**8**

109 . sum mopd02 if mopd3 ==1

Variable	Obs	Mean	Std. Dev.	Min	Max
mopd02	**103**	**8.320388**	**5.080168**	**0**	**21**

110 . sum mopd02 if mopd3 ==2

Variable	Obs	Mean	Std. Dev.	Min	Max
mopd02	**43**	**7.813953**	**4.272034**	**1**	**18**

111 .
112 . sum mopd03 if mopd3 ==0

Variable	Obs	Mean	Std. Dev.	Min	Max
mopd03	**678**	**1.806785**	**1.97012**	**0**	**8**

113 . sum mopd03 if mopd3 ==1

Variable	Obs	Mean	Std. Dev.	Min	Max
mopd03	**85**	**6.058824**	**5.162486**	**0**	**28**

```
114 . sum mopd03 if mopd3 ==2

    Variable |        Obs        Mean    Std. Dev.       Min        Max
-------------+---------------------------------------------------------
      mopd03 |         39     8.74359    4.050458          1         19

115 .
116 . log c
      name:  <unnamed>
       log:  C:\stata_datafolder\20180528_HBC\peerJ_log_supplemental_table1.smcl
  log type:  smcl
 closed on:  23 Oct 2018, 15:59:34
```
