# Supplementary material for: Association of late-onset postpartum depression of mothers with expressive language development during infancy and early childhood: the HBC study
Source: PeerJ. 2019 Mar 6;7:e6566. doi: 10.7717/peerj.6566 (PMC6408909; doi:10.7717/peerj.6566)
Supplement: Supplemental Information 8 [file peerj-07-6566-s008.pdf]

```
-----------------------------------------------------------------------------------------------------------
      name:  <unnamed>
       log:  C:¥stata_datafolder¥20180528_HBC¥SES_codebook_20181217.log
  log type:  text
 opened on:  17 Dec 2018, 15:01:29

. codebook id ptinc0

-----------------------------------------------------------------------------------------------------------
id                                                                          Child's ID (4digits, numeric)
-----------------------------------------------------------------------------------------------------------

                  type:  numeric (float)

                 range:  [1811,5977]                  units:  1
         unique values:  969                      missing .:  0/969

                  mean:   4037.46
              std. dev:   1505.27

           percentiles:        10%       25%       50%       75%       90%
                              1933      2125      4611      5302      5713

-----------------------------------------------------------------------------------------------------------
ptinc0                                                                      Household income prior to birth
-----------------------------------------------------------------------------------------------------------

                  type:  numeric (float)
```

```
            range:  [100,2700]                 units:  1
    unique values:  247                    missing .:  0/969

             mean:   617.607
         std. dev:   283.935

      percentiles:        10%       25%       50%       75%       90%
                          340       440       552       730      1000

. log c
      name:  <unnamed>
       log:  C:¥stata_datafolder¥20180528_HBC¥SES_codebook_20181217.log
  log type:  text
 closed on:  17 Dec 2018, 15:01:29
----------------------------------------------------------------------------------------------------
```
