# Supplementary material for: Association of late-onset postpartum depression of mothers with expressive language development during infancy and early childhood: the HBC study
Source: PeerJ. 2019 Mar 6;7:e6566. doi: 10.7717/peerj.6566 (PMC6408909; doi:10.7717/peerj.6566)
Supplement: Supplemental Information 9 — Analyses adjusted for all of covariates including annual household income. [file peerj-07-6566-s009.pdf]

```
      name:  <unnamed>
       log:  C:\stata_datafolder\20180528_HBC\20181217_SESadded_log.smcl
  log type:  smcl
 opened on:  17 Dec 2018, 22:13:12

1 .
2 . *** Table 1 ***
3 . sum ptinc0
```

Variable	Obs	Mean	Std. Dev.	Min	Max
ptinc0	969	617.6068	283.9349	100	2700

```
4 .
5 . log c
      name:  <unnamed>
       log:  C:\stata_datafolder\20180528_HBC\20181217_SESadded_log.smcl
  log type:  smcl
 closed on:  17 Dec 2018, 22:13:12

      name:  <unnamed>
       log:  C:\stata_datafolder\20180528_HBC\20181217_SESadded_log.smcl
  log type:  smcl
 opened on:  17 Dec 2018, 22:13:12

6 .
7 . *** mopd3 vs MSEL: Table 2 ***
8 . mi est, saving(es): reg mlelt10 i.mopd3 male i.parit3 twin bw ga ptinc0 moag01 moed01 faag01

Multiple-imputation estimates                   Imputations       =         20
Linear regression                               Number of obs     =        969
                                                Average RVI       =     0.0488
                                                Largest FMI       =     0.1044
                                                Complete DF       =        952
DF adjustment:   Small sample                   DF:     min       =     576.67
                                                        avg       =     805.54
                                                        max       =     903.73
Model F test:       Equal FMI                   F(  16,  943.1)   =       3.62
Within VCE type:          OLS                   Prob > F          =     0.0000
```

mlelt10	Coef.	Std. Err.	t	P>|t|	[95% Conf.	Interval]
mopd3						
PPD_Early	.8784054	1.042856	0.84	0.400	-1.168561	2.925372
PPD_Late	.1350416	1.533248	0.09	0.930	-2.874338	3.144422
male	-1.707814	.6319078	-2.70	0.007	-2.948032	-.4675956
parit3						
1	-.1215145	.7115049	-0.17	0.864	-1.518192	1.275163
2+	-1.303159	1.044667	-1.25	0.213	-3.353724	.7474062
twin	-2.569248	1.90124	-1.35	0.177	-6.300714	1.162218
bw	.0026702	.000917	2.91	0.004	.0008703	.00447
ga	.5429833	.2788155	1.95	0.052	-.0044345	1.090401
ptinc0	.0003401	.001239	0.27	0.784	-.0020916	.0027718
moag01	.1689023	.0979225	1.72	0.085	-.0233284	.3611331
moed01	.1909828	.1895137	1.01	0.314	-.1809875	.562953
faag01	-.1940066	.0834724	-2.32	0.020	-.3578421	-.0301712
faed01	-.1758145	.1372275	-1.28	0.200	-.4451363	.0935072
mopsyafdfor0	-.4268611	1.063067	-0.40	0.688	-2.513397	1.659675
mopsyanxfor0	-.9104294	1.724988	-0.53	0.598	-4.296654	2.475796
bf	.0483292	.0531333	0.91	0.363	-.0560026	.152661
_cons	20.48025	10.34822	1.98	0.048	.1554547	40.80504

```
 9 . erase es.ster

10 .
11 . mi est, saving(es): reg mlelt14 i.mopd3 male i.parit3 twin bw ga ptinc0 moag01 moed01 faag01
```

Multiple-imputation estimates		Imputations	=	**20**
Linear regression		Number of obs	=	**969**
		Average RVI	=	**0.1124**
		Largest FMI	=	**0.2046**
		Complete DF	=	**952**
DF adjustment:	**Small sample**	DF: min	=	**290.69**
		avg	=	**606.99**
		max	=	**834.85**
Model F test:	**Equal FMI**	F(**16**, **918.4**)	=	**4.51**
Within VCE type:	**OLS**	Prob > F	=	**0.0000**

mlelt14	Coef.	Std. Err.	t	P>|t|	[95% Conf.	Interval]
mopd3						
PPD_Early	**-.3489003**	**1.128808**	**-0.31**	**0.757**	**-2.564829**	**1.867029**
PPD_Late	**-1.444098**	**1.688266**	**-0.86**	**0.393**	**-4.759154**	**1.870957**
male	**-2.326837**	**.7107944**	**-3.27**	**0.001**	**-3.723159**	**-.930516**
parit3						
1	**.6115509**	**.7596651**	**0.81**	**0.421**	**-.8795271**	**2.102629**
2+	**1.068106**	**1.164886**	**0.92**	**0.360**	**-1.220314**	**3.356526**
twin	**-7.391575**	**2.092095**	**-3.53**	**0.000**	**-11.49912**	**-3.28403**
bw	**.0028624**	**.0010061**	**2.85**	**0.005**	**.0008872**	**.0048377**
ga	**.2775021**	**.3104304**	**0.89**	**0.372**	**-.3325772**	**.8875815**
ptinc0	**-.001012**	**.0013616**	**-0.74**	**0.458**	**-.0036854**	**.0016615**
moag01	**.016709**	**.1069552**	**0.16**	**0.876**	**-.193325**	**.226743**
moed01	**.257825**	**.2076233**	**1.24**	**0.215**	**-.1498215**	**.6654715**
faag01	**-.1375712**	**.0946621**	**-1.45**	**0.147**	**-.3236021**	**.0484596**
faed01	**-.189742**	**.1497345**	**-1.27**	**0.205**	**-.4836642**	**.1041802**
mopsyafdfor0	**1.535893**	**1.252788**	**1.23**	**0.221**	**-.9297924**	**4.001578**
mopsyanxfor0	**-4.198751**	**1.893982**	**-2.22**	**0.027**	**-7.91842**	**-.479083**
bf	**.0763039**	**.0578628**	**1.32**	**0.188**	**-.037353**	**.1899609**
_cons	**34.05232**	**11.31179**	**3.01**	**0.003**	**11.82369**	**56.28096**

```
12 . erase es.ster

13 .
14 . mi est, saving(es): reg mlelt18 i.mopd3 male i.parit3 twin bw ga ptinc0 moag01 moed01 faag01
```

Multiple-imputation estimates		Imputations	=	**20**
Linear regression		Number of obs	=	**969**
		Average RVI	=	**0.0644**
		Largest FMI	=	**0.1228**
		Complete DF	=	**952**
DF adjustment:	**Small sample**	DF: min	=	**507.49**
		avg	=	**759.29**
		max	=	**864.96**
Model F test:	**Equal FMI**	F(**16**, **938.1**)	=	**4.54**
Within VCE type:	**OLS**	Prob > F	=	**0.0000**

mlelt18	Coef.	Std. Err.	t	P>|t|	[95% Conf.	Interval]
mopd3						
PPD_Early	**1.035276**	**1.052294**	**0.98**	**0.326**	**-1.03137**	**3.101922**
PPD_Late	**-3.456846**	**1.529024**	**-2.26**	**0.024**	**-6.458711**	**-.4549804**
male	**-2.930991**	**.6243618**	**-4.69**	**0.000**	**-4.156503**	**-1.70548**
parit3						
1	**-.525465**	**.6945662**	**-0.76**	**0.450**	**-1.888793**	**.8378628**
2+	**-.2038627**	**1.025239**	**-0.20**	**0.842**	**-2.216305**	**1.80858**
twin	**-1.886649**	**1.959963**	**-0.96**	**0.336**	**-5.737289**	**1.963991**

	Coef.	Std. Err.	t	P>|t|	[95% Conf.	Interval]
bw	**.0034686**	**.0008997**	**3.86**	**0.000**	**.0017027**	**.0052346**
ga	**.0986129**	**.2707123**	**0.36**	**0.716**	**-.4327952**	**.630021**
ptinc0	**-.0006823**	**.0012232**	**-0.56**	**0.577**	**-.0030833**	**.0017187**
moag01	**.1070037**	**.0956278**	**1.12**	**0.263**	**-.0807085**	**.2947159**
moed01	**.3674318**	**.1878744**	**1.96**	**0.051**	**-.0013778**	**.7362414**
faag01	**-.1357872**	**.0829556**	**-1.64**	**0.102**	**-.29864**	**.0270657**
faed01	**-.0280238**	**.1364948**	**-0.21**	**0.837**	**-.2959507**	**.2399031**
mopsyafdfor0	**-.8894855**	**1.045423**	**-0.85**	**0.395**	**-2.941455**	**1.162484**
mopsyanxfor0	**-.9837988**	**1.716937**	**-0.57**	**0.567**	**-4.355185**	**2.387587**
bf	**.094631**	**.0510921**	**1.85**	**0.064**	**-.0056558**	**.1949178**
_cons	**32.22546**	**9.960645**	**3.24**	**0.001**	**12.67036**	**51.78056**

```
15 . erase es.ster

16 .
17 . mi est, saving(es): reg mlelt24 i.mopd3 male i.parit3 twin bw ga ptinc0 moag01 moed01 faag01
```

Multiple-imputation estimates		Imputations	=	**20**
Linear regression		Number of obs	=	**969**
		Average RVI	=	**0.0792**
		Largest FMI	=	**0.1177**
		Complete DF	=	**952**
DF adjustment:	**Small sample**	DF: min	=	**525.65**
		avg	=	**722.27**
		max	=	**900.53**
Model F test:	**Equal FMI**	F(**16**, **933.9**)	=	**5.68**
Within VCE type:	**OLS**	Prob > F	=	**0.0000**

mlelt24	Coef.	Std. Err.	t	P>|t|	[95% Conf.	Interval]
mopd3						
PPD_Early	**.8125929**	**1.069294**	**0.76**	**0.448**	**-1.286799**	**2.911985**
PPD_Late	**-5.578102**	**1.590287**	**-3.51**	**0.000**	**-8.7009**	**-2.455305**
male	**-3.564292**	**.6571029**	**-5.42**	**0.000**	**-4.854576**	**-2.274008**
parit3						
1	**-.6009594**	**.7273765**	**-0.83**	**0.409**	**-2.029107**	**.8271883**
2+	**-.742233**	**1.042187**	**-0.71**	**0.477**	**-2.78763**	**1.303164**
twin	**-.0571122**	**1.975359**	**-0.03**	**0.977**	**-3.935809**	**3.821584**
bw	**.0035198**	**.0009278**	**3.79**	**0.000**	**.0016987**	**.0053409**
ga	**-.0073355**	**.2795085**	**-0.03**	**0.979**	**-.5560342**	**.5413633**
ptinc0	**.0000754**	**.0012822**	**0.06**	**0.953**	**-.0024421**	**.0025929**
moag01	**.0840436**	**.0995894**	**0.84**	**0.399**	**-.1114839**	**.2795712**
moed01	**.3775889**	**.1947703**	**1.94**	**0.053**	**-.0048047**	**.7599825**
faag01	**-.1076562**	**.08749**	**-1.23**	**0.219**	**-.2795031**	**.0641908**
faed01	**.1345896**	**.1418526**	**0.95**	**0.343**	**-.1438968**	**.4130761**
mopsyafdfor0	**-1.813335**	**1.069536**	**-1.70**	**0.090**	**-3.912472**	**.2858012**
mopsyanxfor0	**-2.418976**	**1.733091**	**-1.40**	**0.163**	**-5.820816**	**.9828647**
bf	**.1020751**	**.0546375**	**1.87**	**0.062**	**-.0052595**	**.2094097**
_cons	**34.45221**	**10.16066**	**3.39**	**0.001**	**14.50766**	**54.39676**

```
18 . erase es.ster

19 .
20 . mi est, saving(es): reg mlelt32 i.mopd3 male i.parit3 twin bw ga ptinc0 moag01 moed01 faag01
```

Multiple-imputation estimates		Imputations	=	**20**
Linear regression		Number of obs	=	**969**
		Average RVI	=	**0.1211**
		Largest FMI	=	**0.2002**
		Complete DF	=	**952**
DF adjustment:	**Small sample**	DF: min	=	**299.11**
		avg	=	**563.07**
		max	=	**846.63**
Model F test:	**Equal FMI**	F(**16**, **917.8**)	=	**6.92**
Within VCE type:	**OLS**	Prob > F	=	**0.0000**

mlelt32	Coef.	Std. Err.	t	P>|t|	[95% Conf.	Interval]
mopd3						
PPD_Early	.5456742	1.065917	0.51	0.609	-1.547142	2.638491
PPD_Late	-4.680582	1.596651	-2.93	0.004	-7.816535	-1.54463
male	-2.931613	.6485512	-4.52	0.000	-4.204875	-1.65835
parit3						
1	-1.123035	.7485923	-1.50	0.134	-2.594168	.3480982
2+	-1.962061	1.070483	-1.83	0.067	-4.064024	.1399028
twin	1.299949	2.011473	0.65	0.518	-2.652011	5.251909
bw	.0021563	.000949	2.27	0.023	.0002928	.0040199
ga	.2420647	.2992962	0.81	0.419	-.3467933	.8309226
ptinc0	.002623	.0013186	1.99	0.047	.0000316	.0052143
moag01	.0487273	.1000758	0.49	0.626	-.1477954	.2452501
moed01	.4694351	.2050442	2.29	0.023	.0661948	.8726755
faag01	-.0458531	.0870128	-0.53	0.598	-.2167582	.1250519
faed01	.3189691	.1439342	2.22	0.027	.0362747	.6016635
mopsyafdfor0	-2.438037	1.069982	-2.28	0.023	-4.538166	-.3379085
mopsyanxfor0	-4.173744	1.765558	-2.36	0.018	-7.640843	-.7066444
bf	.0774798	.0535329	1.45	0.148	-.0276409	.1826006
_cons	22.34709	10.99578	2.03	0.043	.7081977	43.98598

```
21 . erase es.ster

22 .
23 . mi est, saving(es): reg mlelt40 i.mopd3 male i.parit3 twin bw ga ptinc0 moag01 moed01 faag01
```

```
Multiple-imputation estimates                     Imputations       =         20
Linear regression                                 Number of obs     =        969
                                                  Average RVI       =     0.0800
                                                  Largest FMI       =     0.1023
                                                  Complete DF       =        952
DF adjustment:   Small sample                     DF:     min       =     584.97
                                                          avg       =     707.44
                                                          max       =     802.91
Model F test:       Equal FMI                     F(  16,  932.4)   =       9.25
Within VCE type:          OLS                     Prob > F          =     0.0000
```

mlelt40	Coef.	Std. Err.	t	P>|t|	[95% Conf.	Interval]
mopd3						
PPD_Early	-1.152732	1.076555	-1.07	0.285	-3.266207	.9607425
PPD_Late	-5.489517	1.590696	-3.45	0.001	-8.61241	-2.366624
male	-3.51491	.6581649	-5.34	0.000	-4.807027	-2.222792
parit3						
1	-1.659444	.7386782	-2.25	0.025	-3.109921	-.2089666
2+	-3.265772	1.071161	-3.05	0.002	-5.368466	-1.163079
twin	2.96945	2.017911	1.47	0.142	-.9937824	6.932683
bw	.0029577	.0009568	3.09	0.002	.0010792	.0048363
ga	.2860102	.2831122	1.01	0.313	-.2697805	.841801
ptinc0	.0028425	.0012868	2.21	0.027	.0003163	.0053688
moag01	.137463	.1003057	1.37	0.171	-.0594562	.3343821
moed01	.5883871	.1998392	2.94	0.003	.1959109	.9808632
faag01	-.0888677	.0871392	-1.02	0.308	-.259963	.0822275
faed01	.3303654	.1444708	2.29	0.023	.0466971	.6140337
mopsyafdfor0	.1136272	1.110316	0.10	0.919	-2.066473	2.293728
mopsyanxfor0	-.984245	1.755207	-0.56	0.575	-4.42958	2.46109
bf	.1274077	.0538403	2.37	0.018	.0217053	.2331101
_cons	15.71294	10.33777	1.52	0.129	-4.581343	36.00721

```
24 . erase es.ster

25 .
26 . log c
      name:  <unnamed>
       log:  C:\stata_datafolder\20180528_HBC\20181217_SESadded_log.smcl
  log type:  smcl
 closed on:  17 Dec 2018, 22:13:26
-------------------------------------------------------------------------------
-------------------------------------------------------------------------------
      name:  <unnamed>
       log:  C:\stata_datafolder\20180528_HBC\20181217_SESadded_log.smcl
  log type:  smcl
 opened on:  17 Dec 2018, 22:13:30

27 .
28 . *** Table 3 ***
29 . mixed mlelt c.t##c.t##i.mopd3 male i.parit3 twin bw ga ptinc0 ///
> moag01 moed01 faag01 faed01 mopsyafdfor0 mopsyanxfor0 c.bf ///
> if t==10 | t==14 | t==18 | t==24 | t==32 | t==40 || id: t, cov(uns) vce(cluster moid) var

Performing EM optimization:

Performing gradient-based optimization:

Iteration 0:   log pseudolikelihood = -18677.317
Iteration 1:   log pseudolikelihood = -18677.315
Iteration 2:   log pseudolikelihood = -18677.315

Computing standard errors:

Mixed-effects regression                        Number of obs     =      5,243
Group variable: id                              Number of groups  =        969

                                                Obs per group:
                                                              min =          1
                                                              avg =        5.4
                                                              max =          6

                                                Wald chi2(22)     =     167.98
Log pseudolikelihood = -18677.315               Prob > chi2       =     0.0000

                                  (Std. Err. adjusted for 880 clusters in moid)
```

mlelt	Coef.	Robust Std. Err.	z	P>|z|	[95% Conf.	Interval]
t	.0817718	.0707555	1.16	0.248	-.0569063	.2204499
c.t#c.t	-.0004497	.0013209	-0.34	0.734	-.0030386	.0021392
mopd3						
PPD_Early	-.0501373	2.241195	-0.02	0.982	-4.442798	4.342523
PPD_Late	6.517835	3.792122	1.72	0.086	-.9145871	13.95026
mopd3#c.t						
PPD_Early	.1082812	.1977562	0.55	0.584	-.2793139	.4958763
PPD_Late	-.7397687	.3510081	-2.11	0.035	-1.427732	-.0518055
mopd3#c.t#c.t						
PPD_Early	-.0033417	.003816	-0.88	0.381	-.010821	.0041376
PPD_Late	.0110893	.0068995	1.61	0.108	-.0024335	.0246122
male	-2.714241	.4414514	-6.15	0.000	-3.57947	-1.849012
parit3						
1	-.4417205	.5015902	-0.88	0.379	-1.424819	.5413782
2+	-.7493154	.6775878	-1.11	0.269	-2.077363	.5787323
twin	-1.836303	1.499684	-1.22	0.221	-4.775629	1.103024
bw	.0030161	.0006424	4.69	0.000	.001757	.0042753
ga	.2483747	.1927844	1.29	0.198	-.1294758	.6262252
ptinc0	.0004511	.0008032	0.56	0.574	-.0011232	.0020254

moag01	.1048003	.0720378	1.45	0.146	-.0363911	.2459918
moed01	.3380313	.1256603	2.69	0.007	.0917417	.5843209
faag01	-.1367185	.0603388	-2.27	0.023	-.2549804	-.0184565
faed01	.0260802	.0982551	0.27	0.791	-.1664962	.2186567
mopsyafdfor0	-.5939472	.8218502	-0.72	0.470	-2.204744	1.01685
mopsyanxfor0	-2.271809	1.297086	-1.75	0.080	-4.814051	.2704325
bf	.0829768	.0359888	2.31	0.021	.01244	.1535136
_cons	25.81529	7.246515	3.56	0.000	11.61239	40.0182

Random-effects Parameters	Estimate	Robust Std. Err.	[95% Conf.	Interval]
**id**: Unstructured				
var(t)	.0896732	.0089444	.0737496	.1090349
var(_cons)	67.45819	5.745698	57.08661	79.71409
cov(t,_cons)	-1.726095	.2065659	-2.130957	-1.321233
var(Residual)	47.62708	1.39883	44.96284	50.44918

```
30 .
31 . mixed mlelt c.t##i.mopd3 male i.parit3 twin bw ga ptinc0 ///
> moag01 moed01 faag01 faed01 mopsyafdfor0 mopsyanxfor0 c.bf ///
> if t==10 | t==14 | t==18 | t==24 | t==32 | t==40 || id: t, cov(uns) vce(cluster moid) var

Performing EM optimization:

Performing gradient-based optimization:

Iteration 0:   log pseudolikelihood = -18680.064
Iteration 1:   log pseudolikelihood = -18680.062
Iteration 2:   log pseudolikelihood = -18680.062

Computing standard errors:

Mixed-effects regression                        Number of obs     =      5,243
Group variable: id                              Number of groups  =        969

                                                Obs per group:
                                                              min =          1
                                                              avg =        5.4
                                                              max =          6

                                                Wald chi2(19)     =     165.22
Log pseudolikelihood = -18680.062               Prob > chi2       =     0.0000

                                   (Std. Err. adjusted for 880 clusters in moid)
```

mlelt	Coef.	Robust Std. Err.	z	P>|z|	[95% Conf.	Interval]
t	.059393	.0156299	3.80	0.000	.028759	.0900269
mopd3						
PPD_Early	1.633861	1.140133	1.43	0.152	-.6007575	3.86848
PPD_Late	.9033042	1.829348	0.49	0.621	-2.682151	4.48876
mopd3#c.t						
PPD_Early	-.0577733	.0393359	-1.47	0.142	-.1348703	.0193237
PPD_Late	-.1874651	.0615341	-3.05	0.002	-.3080696	-.0668605
male	-2.718403	.4418076	-6.15	0.000	-3.58433	-1.852475
parit3						
1	-.4360222	.5019212	-0.87	0.385	-1.41977	.5477252
2+	-.7456034	.6775418	-1.10	0.271	-2.073561	.5823542
twin	-1.814682	1.520308	-1.19	0.233	-4.794431	1.165066
bw	.0030209	.0006424	4.70	0.000	.0017618	.00428
ga	.2485748	.1929178	1.29	0.198	-.1295371	.6266867
ptinc0	.0004511	.0008032	0.56	0.574	-.0011232	.0020253

```
      moag01 |   .1038414   .0720745     1.44   0.150     -.037422    .2451047
      moed01 |   .3383117   .1257898     2.69   0.007     .0917682    .5848551
      faag01 |  -.1365739   .0603479    -2.26   0.024    -.2548536   -.0182941
      faed01 |   .0263309   .0982903     0.27   0.789    -.1663146    .2189763
mopsyafdfor0 |  -.5890064   .8212951    -0.72   0.473    -2.198715    1.020702
mopsyanxfor0 |  -2.284554   1.297302    -1.76   0.078    -4.827218    .2581105
          bf |   .0828767   .0359911     2.30   0.021     .0123355    .1534179
       _cons |   26.04012   7.208838     3.61   0.000     11.91106    40.16918
------------------------------------------------------------------------------

------------------------------------------------------------------------------
                             |               Robust
  Random-effects Parameters  |   Estimate   Std. Err.     [95% Conf. Interval]
-----------------------------+------------------------------------------------
id: Unstructured             |
                      var(t) |   .0896174   .0089549      .0736778    .1090054
                  var(_cons) |   67.50925   5.759997      57.11332    79.79747
                cov(t,_cons) |  -1.726587    .206935     -2.132173   -1.321002
-----------------------------+------------------------------------------------
               var(Residual) |   47.69429   1.405312      45.01795    50.52974
------------------------------------------------------------------------------

32 .
33 . log c
      name:  <unnamed>
       log:  C:\stata_datafolder\20180528_HBC\20181217_SESadded_log.smcl
  log type:  smcl
 closed on:  17 Dec 2018, 22:13:57
-------------------------------------------------------------------------------------
-------------------------------------------------------------------------------------
      name:  <unnamed>
       log:  C:\stata_datafolder\20180528_HBC\20181217_SESadded_log.smcl
  log type:  smcl
 opened on:  17 Dec 2018, 22:13:57

34 .
35 . *** Supplemental table 1 ***
36 . sum ptinc0 if mopd3 ==0

    Variable |        Obs        Mean    Std. Dev.       Min        Max
-------------+---------------------------------------------------------
      ptinc0 |        823    623.8834    295.0544        100       2700

37 . sum ptinc0 if mopd3 ==1

    Variable |        Obs        Mean    Std. Dev.       Min        Max
-------------+---------------------------------------------------------
      ptinc0 |        103    593.8932    228.2142        220       1850

38 . sum ptinc0 if mopd3 ==2

    Variable |        Obs        Mean    Std. Dev.       Min        Max
-------------+---------------------------------------------------------
      ptinc0 |         43    554.2791     146.642        300        900

39 .
40 . log c
      name:  <unnamed>
       log:  C:\stata_datafolder\20180528_HBC\20181217_SESadded_log.smcl
  log type:  smcl
 closed on:  17 Dec 2018, 22:13:57
-------------------------------------------------------------------------------------
```
